# Silicon Nitride, a Bioceramic for Bone Tissue Engineering: A Reinforced Cryogel System With Antibiofilm and Osteogenic Effects

**DOI:** 10.3389/fbioe.2021.794586

**Published:** 2021-12-15

**Authors:** Seunghun S. Lee, Leanid Laganenka, Xiaoyu Du, Wolf-Dietrich Hardt, Stephen J. Ferguson

**Affiliations:** ^1^ Department of Health Sciences and Technology, Institute for Biomechanics, ETH Zurich, Zurich, Switzerland; ^2^ Department of Biology, Institute of Microbiology, ETH Zurich, Zurich, Switzerland

**Keywords:** silicon nitride, cryogel, bone tissue engineering, biomaterials, antibacterial, osteogenic, bioreactor

## Abstract

Silicon nitride (SiN [Si_3_N_4_]) is a promising bioceramic for use in a wide variety of orthopedic applications. Over the past decades, it has been mainly used in industrial applications, such as space shuttle engines, but not in the medical field due to scarce data on the biological effects of SiN. More recently, it has been increasingly identified as an emerging material for dental and orthopedic implant applications. Although a few reports about the antibacterial properties and osteoconductivity of SiN have been published to date, there have been limited studies of SiN-based scaffolds for bone tissue engineering. Here, we developed a silicon nitride reinforced gelatin/chitosan cryogel system (SiN-GC) by loading silicon nitride microparticles into a gelatin/chitosan cryogel (GC), with the aim of producing a biomimetic scaffold with antibiofilm and osteogenic properties. In this scaffold system, the GC component provides a hydrophilic and macroporous environment for cells, while the SiN component not only provides antibacterial properties and osteoconductivity but also increases the mechanical stiffness of the scaffold. This provides enhanced mechanical support for the defect area and a better osteogenic environment. First, we analyzed the scaffold characteristics of SiN-GC with different SiN concentrations, followed by evaluation of its apatite-forming capacity in simulated body fluid and protein adsorption capacity. We further confirmed an antibiofilm effect of SiN-GC against *Escherichia coli* (*E. coli*) and *Staphylococcus aureus* (*S. aureus*) as well as enhanced cell proliferation, mineralization, and osteogenic gene upregulation for MC3T3-E1 pre-osteoblast cells. Finally, we developed a bioreactor to culture cell-laden scaffolds under cyclic compressive loading to mimic physiological conditions and were able to demonstrate improved mineralization and osteogenesis from SiN-GC. Overall, we confirmed the antibiofilm and osteogenic effect of a silicon nitride reinforced cryogel system, and the results indicate that silicon nitride as a biomaterial system component has a promising potential to be developed further for bone tissue engineering applications.

## Introduction

Bone is a complex tissue that continuously undergoes dynamic biological remodeling to maintain homeostasis. However, healing in large and critical defect areas is often impaired, leading to inferior bone regeneration and extended hospitalization (Bose et al., 2012; Zhang et al., 2016; Annamalai et al., 2019; Koons et al., 2020). Along with this, the increasing number of bone fractures and orthopedic-related injuries due to an exponential growth of the elderly population has prompted researchers to explore bone tissue engineering to address these issues (Bose et al., 2012; Gong et al., 2015; Longoni et al., 2018). Many therapeutic strategies have been suggested to promote bone regeneration, including scaffolds (Lin et al., 2019; Zhang et al., 2019; Zhou et al., 2019), stem cells (Annamalai et al., 2019; Kim et al., 2019; Kim et al., 2020), and osteogenic factors (Naskar et al., 2017; Lee et al., 2020; Amirthalingam et al., 2021; Lee et al., 2021b). More recently, biomaterial scaffolds that can promote bone tissue repair on their own, without the need for delivering cells, have emerged as a potentially powerful paradigm for bone tissue engineering, due to their promising advantages of reduced cost and fewer translational barriers than other regenerative medicine strategies, such as cell-based therapy (Christman, 2019; Montoya et al., 2021). Thus, the development of scaffolds with appropriate biomaterials became one of the key success paths for bone tissue engineering (Bose et al., 2012; Kim et al., 2020).

As one of many potential solutions, silicon nitride (SiN [Si_3_N_4_]), a synthetic non-oxide ceramic with high stiffness, strength, and fracture resistance, has been recently proposed as a promising biomaterial for orthopedic applications (Ishikawa et al., 2017; Lal et al., 2018; Sainz et al., 2020). According to the studies, SiN has several key advantages of biocompatibility, hydrophilicity, stable mechanical properties, and excellent imaging across all modalities, such as computed tomography (CT) and magnetic resonance imaging (MRI), which are indispensable elements for scaffolds (Bal and Rahaman, 2012; Webster et al., 2012; Pezzotti et al., 2017b; Rambo, 2018). In addition, antibacterial and osteoconductive properties of SiN make it a unique biomaterial for bone tissue engineering applications by preventing infection after surgery and promoting bone tissue regeneration simultaneously (Ishikawa et al., 2017; Lee et al., 2021a). Osteogenic effects of SiN were confirmed in several *in vitro* tests with different cell types, such as human osteosarcoma SaOS-2 cells (Pezzotti et al., 2016; Pezzotti et al., 2017b; Zanocco et al., 2019), mouse bone marrow stromal cells (BMSCs) (Pezzotti et al., 2017a), and human BMSCs (Amaral et al., 2002). The effectiveness of SiN was further confirmed in several *in vivo* studies using a goat lumbar interbody fusion model (Kersten et al., 2019), a rat calvarial defect model (Webster et al., 2012), and a murine tibial implant model (Ishikawa et al., 2017). However, the brittleness and lack of resilience, common disadvantages of ceramic biomaterials, make it problematic to use SiN alone as a scaffold. As the use of SiN in medical fields is a fairly recent development, to date, there are scarce data on SiN for bone tissue engineering applications. Particularly, to the best of our knowledge, there has not been a study of scaffolds using SiN combined with a hydrogel which can provide a hydrophilic, biocompatible, and porous structure similar to the extracellular matrix (ECM) (Yu and Ding, 2008; Yue et al., 2020).

**FIGURE 1:**
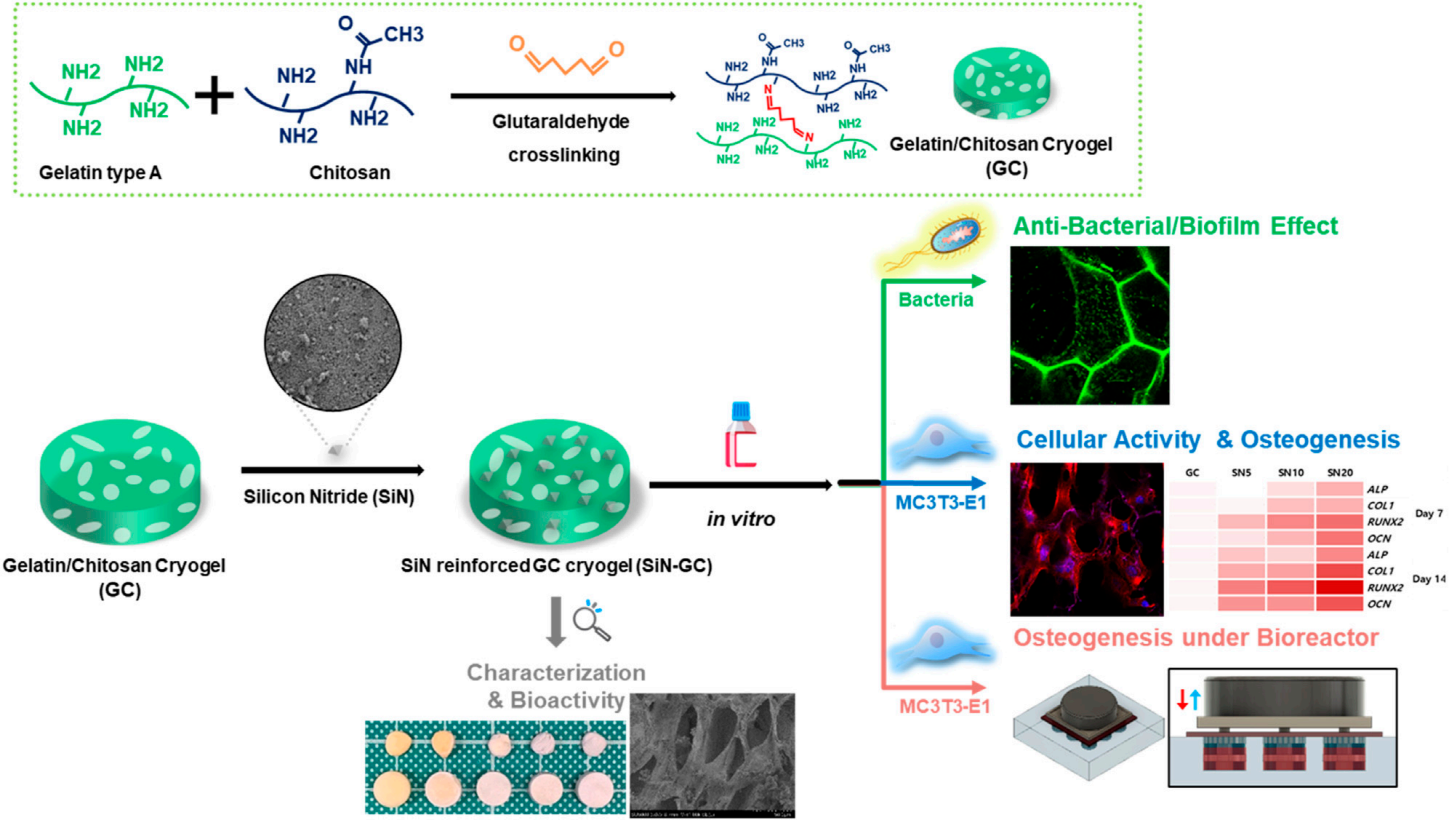
Schematic illustration of silicon nitride reinforced cryogel (SiN-GC). First, gelatin/chitosan cryogel (GC) was synthesized via cross-linking between gelatin and chitosan by the glutaraldehyde-mediated cryogelation process, and silicon nitride (SiN) was used to reinforce GC to fabricate SiN-GC. After analysis of the characteristics and bioactivity of SiN-GC, *in vitro* experiments were carried out to investigate biological properties such as antibacterial effects, cellular response, mineralization, and osteogenesis under both static and cyclic loading conditions.

In this study, we developed a SiN reinforced gelatin/chitosan cryogel system (SiN-GC) by loading SiN microparticles into a macroporous gelatin/chitosan cryogel (GC), a type of hydrogel with a highly interconnected and macroporous structure, formed by lyophilizing ice crystals during cryogelation at a subzero temperature, that has been fabricated in a previous study (Lee et al., 2020) (Figure 1). Such a biomimetic scaffold provides an optimal environment for cells, potentially combined with antibacterial and osteogenic effects. In this scaffold system, the GC component provides a biocompatible, hydrophilic, and macroporous environment for cells, while the SiN component not only may provide antibacterial properties and osteoconductivity but also increase the mechanical stiffness of the scaffold, to provide enhanced mechanical support for the defect area and a better osteogenic environment (Engler et al., 2006; Chen et al., 2015; Zhang et al., 2018). In this study, we focused on three aims to study the potential of SiN-GC for bone tissue engineering applications: 1) fabrication of SiN-GC to overcome the limitations of SiN and GC, 2) investigation of the functionalities of SiN-GC such as antibiofilm and osteogenic effects, and 3) evaluation of the osteogenic profile of the SiN-GC cryogel system under simulated physiological cyclic loading conditions in a bioreactor.

## Materials and Methods

### Fabrication of Gelatin/Chitosan Cryogel and Silicon Nitride Reinforced Cryogel

First, gelatin/chitosan cryogel (GC) was polymerized *via* a glutaraldehyde cross-linking reaction as described in a previous study (Lee et al., 2020). Briefly, 1% (w/v) of type A gelatin (Sigma-Aldrich, G1890) and 0.3% (w/v) of chitosan (Merck Millipore, 375,095) were dissolved in 1% acetic acid solution (Merck, 100063). Then, 1% (w/v) glutaraldehyde (Sigma-Aldrich, 340855) was used as the cross-linker for cryogelation, and the used volume of 1% glutaraldehyde for GC was one-fourth of the mixed gelatin/chitosan solution. After mixing all the solutions homogenously, 200 μL of the precursor solution was put in a pre-cooled cylindrical cryogel mold and placed at −20°C overnight to induce cryogelation. After cryogelation, cryogels were lyophilized for a minimum of 6 h to remove ice crystals. After lyophilization, GC cryogels were soaked and fully swollen with distilled water (DW) until use in further experiments.

For the silicon nitride reinforced cryogel (SiN-GC), medical-grade silicon nitride microparticles (SiN) (MC^2^ silicon nitride, SINTX Technologies, United States; median particle size: 0.279 μm of diameter) were dispersed in DW homogenously to obtain 1, 5, 10, and 20% (w/v) of SiN solution for each SiN-GC group (SN1, SN5, SN10, and SN20, respectively). Then, a GC cryogel was placed in 1 ml of SiN solution in a microtube and vortexed at 1400 RPM for 2 h in a thermomixer (Eppendorf, ThermoMixer). After vortexing, SiN-GCs were dipped in clean DW and vortexed at 500 RPM for 1 h to wash out extra SiN on the surface of the cryogels. For *in vitro* experiments, the SiN-GCs were soaked with PBS and sterilized by UV irradiation for 3 h.

### Silicon Nitride Loading Efficiency Measurement

The loading efficiency of SiN into GC cryogels was assessed as described in the previous study (Kim et al., 2019). Briefly, SiN-GCs were digested in Papain solution (Sigma-Aldrich, P3125) at 60°C. After 24 h, the digested solution was frozen at −20°C and lyophilized to remove water. The weight of the remaining SiN was then measured to determine the loading efficiency.

### Scanning Electron Microscopy

GC and SiN-GC cryogels were frozen, lyophilized, and cut longitudinally to access the cross section. The samples were fixed on metal stubs with carbon tape and coated with platinum/palladium (80/20) sputtering (CCU-010, Safematic). Field emission scanning electron microscopy (FE-SEM) (SEM SU5000, Hitachi) was used to capture the microstructure, distribution of SiN microparticles, and cross sections of the scaffolds at 3 kV. For energy-dispersive X-ray spectroscopy (EDS) analysis to identify ion deposited on the scaffold, FE-SEM (JSM-7100F, JEOL) was used at 8 kV and live time of 30 s.

### Mechanical Test

For compressive mechanical testing, all tested scaffolds were produced in a cylindrical shape, and the dimensions were measured with a digital caliper for precise calculation of elastic modulus. Prior to the testing, the scaffolds were fully swollen with distilled water. Compressive mechanical testing was performed using an electrodynamic material testing machine (Instron, ElectroPuls E10000). An unconfined quasi-static compression was performed between two parallel smooth plates, at a displacement rate of 1 mm/min. After testing, the elastic modulus was calculated from the linear region of the stress–strain curve.

### Swelling Ratio and Interconnected Porosity Measurement

The lyophilized weight of GC and SiN-GC groups (SN1, SN5, SN10, and SN20) was measured after fabrication, freezing, and lyophilization. The lyophilized scaffolds were submerged in PBS at room temperature until the scaffolds were fully swollen. The weights of fully swollen scaffolds were measured to calculate the swelling ratio:

$$\text{Swelling ratio}\left(\text{Q}\right)=\left({\text{W}}_{\text{s}}/{\text{W}}_{\text{l}}\right)\text{×}100,$$

where W_s_ is the weight of fully swollen samples and W_l_ is the weight of the lyophilized samples.

Interconnected porosity was measured as previously described (Lee et al., 2020). First, the scaffolds were submerged in PBS, and the weights of swollen scaffolds were measured. Then, the swollen scaffolds were dehydrated with Kimwipes to completely remove water. The weights of the dehydrated scaffolds were measured to calculate the percentage of interconnected porosity:

$$\text{\% Interconnected porosity }=\left({\text{W}}_{\text{s}}{-\text{W}}_{\text{d}}\right)/{\text{W}}_{\text{s}}\text{×}100\text{,}$$

where W_s_ is the weight of fully swollen cryogels and W_d_ is the weight of dehydrated cryogels.

### Enzyme-Mediated Degradation of Cryogel

To measure enzyme-mediated degradation of the cryogel, weights of fully swollen GC and SiN-GC cryogels were measured (initial weight, W_i_). The cryogels were incubated in 24-well plates filled with 0.25% trypsin—EDTA (ThermoFisher, 25200056) solution at 37°C for 60 days. At every time point, the weights of samples were measured to calculate the degree of degradation:

$$\text{Degree of mass remaining}\left(\text{\%}\right)=100-(\left({\text{W}}_{\text{i}}{-\text{W}}_{\text{dg}}\right)/{\text{W}}_{\text{i}}\text{×}100)\text{,}$$

where W_i_ is the initial weight of samples before degradation and W_dg_ is the weight of samples after degradation.

### Apatite Formation of Scaffolds in Simulated Body Fluid Immersion and ICP Analysis

Fabricated GC, SN5, SN10, and SN20 scaffolds were submerged with 1 ml of SBF solution (58.43 g NaCl, 2.77 g CaCl_2_, and 1.39 g of NaH_2_PO_4_·H_2_O per liter) and incubated at 37°C for 1 and 2 weeks (Kwon et al., 2018). The scaffolds and remaining SBF solutions were subsequently collected, and the scaffolds were washed with distilled water (DW) and lyophilized. Apatite formation was analyzed by SEM and EDS.

For inductively coupled plasma mass spectrometry (ICP-MS), collected SBF solutions were used to measure the remaining amount of P^3+^ and Ca^2+^ ions in SBF by using ICP-MS (iCAP RQ ICP-MS, ThermoFisher).

### Protein Adsorption Analysis

To measure the protein adsorption capacity of GC, SN5, SN10, and SN20 cryogels, each scaffold was placed in a 24-well plate filled with 1 ml of 0.1 mg/ml or 1 mg/ml bovine serum albumin (BSA) solutions for 1 h at room temperature. After collecting the scaffolds, a Bradford protein assay (ThermoFisher, 23200) was used to measure the protein adsorbed on the scaffolds by following the manufacturer’s protocol. For the protein adsorption experiment with complete medium, scaffolds were submerged in DMEM/F-12 (ThermoFisher, 31330038) with 10% fetal bovine serum (FBS) (ThermoFisher, 26140079) and incubated for 1 h at room temperature. Then, the scaffolds were collected, and the protein adsorbed was measured with the Bradford protein assay in the same manner.

### Hemolysis Rate Testing

The blood compatibility of the scaffolds was evaluated by hemolysis rate testing. Pig blood was obtained from a city of Zurich authorized butcher shop. The whole blood was centrifuged (2000 rpm, 5 min) to obtain the red blood cells. Collected red blood cells were diluted using PBS (1% v/v), and each scaffold was placed in a 24-well plate filled with 1 ml of the above prepared solution for 2 h. After taking out the scaffolds, the solution was centrifuged at 2000 rpm for 5 min, and the absorbance of the supernatant was measured at 540 nm. The PBS group was used as a negative control group, while the distilled water (DW) group was used as a positive control group. After the absorbance measurement, the hemolysis rate was calculated with the following equation: Hemolysis rate (%) = (OD_sample_ − OD_negative_)/(OD_positive_ − OD_negative_) x 100%.

### Antibiofilm and Antibacterial Activity Analysis

The antibiofilm and antibacterial activities of the SiN-GC scaffolds were evaluated using *E. coli* W3110 (Serra et al., 2013) and *S. aureus* [Hardt Lab strain collection, isolated from mice harboring low complexity microbiota (Stecher et al., 2010)]. Bacterial overnight cultures were grown in Lysogeny broth (LB) medium (10 g tryptone and 5 g NaCl per liter) at 37°C with shaking. The cultures were then diluted 1:100 in 1 ml fresh LB, followed by incubation under static conditions in 24-well microtiter plates (TPP, Switzerland, 92024) for 8 and 32 h at 37°C. At the end of the culture period, the samples were washed in PBS buffer (8 g NaCl, 0.2 g KCl, 1.44 g Na_2_HPO_4_, and 0.24 g KH_2_PO_4_ per liter) to remove loosely adherent bacteria. The amount of remaining attached bacteria was determined using a PrestoBlue assay (ThermoFisher, P50200) by following the manufacturer’s protocol. Additionally, OD_600_ of planktonic cultures was measured with an Ultrospec 10 spectrophotometer (Biochrom, Great Britain).

To allow imaging of bacterial biofilms, *E. coli* cells were transformed with pM965 plasmids constitutively expressing GFPmut2 under control of *rpsM* promoter (Stecher et al., 2004). The biofilms were visualized with confocal laser scanning microscopy (CLSM) (Zeiss, LSM 780 upright).

### Live and Dead Assay and Proliferation Rate

5 × 10^4^ mouse pre-osteoblast cells (MC3T3-E1, obtained from the University of Zurich, Switzerland) were seeded onto the scaffolds. After 2 h of attachment, cells were cultured in growth medium (GM) composed of MEM-α without ascorbic acid (ThermoFisher, A1049001), 10% fetal bovine serum (ThermoFisher, 26140079), and 1% antibiotic–antimycotic (ThermoFisher, 15240062). After 2 days of incubation, cells were stained for 30 min in 0.5 µL/ml calcein-AM and 2 µL/ml ethidium homodimer-1 from the Live/Dead Assay Kit (ThermoFisher, L3224). Then, the cells were visualized with confocal laser scanning microscopy (Zeiss, LSM 780 upright), and viability was calculated as the number of live cells divided by the total number of cells. For the cell proliferation assay, 5 × 10^3^ cells were seeded onto the scaffolds, and a PrestoBlue assay kit (ThermoFisher, P50200) was utilized by following the standard protocol. Briefly, after the attachment of cells, the GM was changed to the assay medium containing 10% of PrestoBlue solution. After 30 min of incubation, the medium was changed to the fresh GM, and the assay medium was collected, and its fluorescence was analyzed with a microplate reader (Tecan, Infinite 200 Pro) at an excitation wavelength of 560 nm and emission of 590 nm. The same procedure was performed on days 0, 2, 4, 6, 8, 10, and 13, and the percentage of reduction was calculated for the proliferation rate.

### Actin/DAPI Staining and Analysis

3 × 10^4^ MC3T3-E1 cells were seeded onto the scaffolds and cultured for 3 days in GM. Then, actin and cell nuclei were stained using Alexa Fluor 568 Phalloidin (ThermoFisher, A12380) and DAPI (ThermoFisher, 62247) by following the manufacturer’s protocol. Briefly, after washing with PBS, samples were fixed in 4% paraformaldehyde (PFA) in PBS for 15 min. After washing with PBS, the cells were permeabilized using 0.1% Triton X-100 in PBS for 15 min, followed by incubation in blocking buffer (0.1% Triton X-100, 1% BSA in PBS) for 45 min. For immunofluorescence staining of actin, samples were stained with fluorescent phalloidin staining solution for 60 min and rinsed three times with PBS. For DAPI staining, the samples were stained with the DAPI solution for 10 min and rinsed five times with PBS to remove excess staining solution. Finally, the samples were visualized with CLSM, and actin length, cell area, and fluorescence intensity were measured and analyzed using ZEN software (Zen 3.0 Blue, Zeiss).

### *In Vitro* Osteogenic Differentiation

Osteogenic medium (OM) was prepared by adding 100 nM of dexamethasone (Sigma-Aldrich, D4902), 10 mM of glycerol-2-phosphate disodium salt hydrate (Sigma-Aldrich, G9422), and 50 µg/ml of L-ascorbic acid (Sigma-Aldrich, A92902) to GM. For 3D cell culture, MC3T3-E1 was seeded on the scaffolds, and cells were cultured in OM at 37°C. For 2D cell culture, OM was used after the cells reached confluence with GM in the plate. Alkaline phosphatase (ALP), Alizarin red S (ARS) staining, and real-time quantitative polymerase chain reaction (RT-qPCR) were performed after 7 and 14 days of culture to determine mineralization and osteogenic differentiation.

### Bioreactor and Cyclic Loading Setup

All the components for the bioreactor such as polycarbonate well plate, flexible membranes, and metal shuttles (CellScale, Canada) were autoclaved for sterilization prior to cyclic loading. The bioreactor setup is schematically illustrated in Supplementary Figures S4A,B. Briefly, the scaffolds were loaded into the center of each well. Each well was then filled with 1 ml of OM. The shuttles were placed on top of the scaffolds, and a flexible membrane was used to cover the well to minimize contamination from outside. Then, a magnet was placed at the center of each well, and a metal plate was used as a connecting plate between the well and the electrodynamic testing machine (Instron, ElectroPuls E10000). The well plate was placed in a glass container filled with distilled water kept at a constant temperature of 37°C by a hotplate (VWR, United States). Cyclic monoaxial compression was applied at a frequency of 1 Hz, strain of 10%, and duration of 1 h/day. Loading was performed every day until sample collection for ARS or RT-qPCR.

### ALP and ARS Staining

After 7 days of osteogenic induction, ALP staining (Sigma-Aldrich, 85L-2) was performed in the 2D cell culture by following the standard protocol (Lee et al., 2020). For calcium deposition, the ARS kit (ScienCell Research, 0223) was used (Lee et al., 2020) after 7, 14, and 21 days of osteogenic induction. Cells were fixed in 4% PFA (Santa Cruz Biotech, 281692) for 15 min and washed three times with distilled water. Then, the samples were stained with 2% ARS solution for 30 min at room temperature and washed with distilled water until excess staining agents were removed. The amount of mineral content was measured by eluting the ARS with 10% cetylpyridinium chloride (Sigma-Aldrich, C0732), and the absorbance was measured with a microplate reader (Tecan, Infinite 200 Pro) at a wavelength of 570 nm.

### Real-Time Quantitative PCR Analysis

After 7 and 14 days of incubation, MC3T3-E1 cell-laden scaffolds were mixed with 1 ml of TRIzol (ThermoFisher, 15596026) and homogenized using a Polytron homogenizer (PT2500E). Then 0.2 ml of chloroform was added and mixed homogenously. After 5 min of incubation at room temperature, the samples were centrifuged at 13,000 g for 10 min at 4°C. The upper aqueous phase was transferred into a new microtube and mixed with the same volume of 70% RNAse-free ethanol. The mixed solution was processed with the RNeasy Plus Mini Kit (Qiagen Inc., United States), following the manufacturer’s protocol. RT-qPCR was performed to confirm osteogenic gene expression levels by using TaqMan gene expression assays with the following probe/primer combinations: *GAPDH*, *Mm99999915_g1*; *ALP*, *Mm00475834_m1*; *COL1*, *Mm00801666_g1*; *RUNX2*, *Mm00501578_m1*; and *OCN*, *Mm03413826_mH* (ThermoFisher Scientific, United States).

### Statistical Analysis

All experiments were performed at least in triplicate, and all data were analyzed as mean ± SD. For statistical analysis, one-way ANOVA was performed followed by Tukey’s post hoc test, and statistical significance was considered by *p*-value: *p < 0.05, **p < 0.01, and ***p < 0.005.

## Results

### Fabrication of Silicon Nitride Reinforced Gelatin/Chitosan Cryogel

**FIGURE 2:**
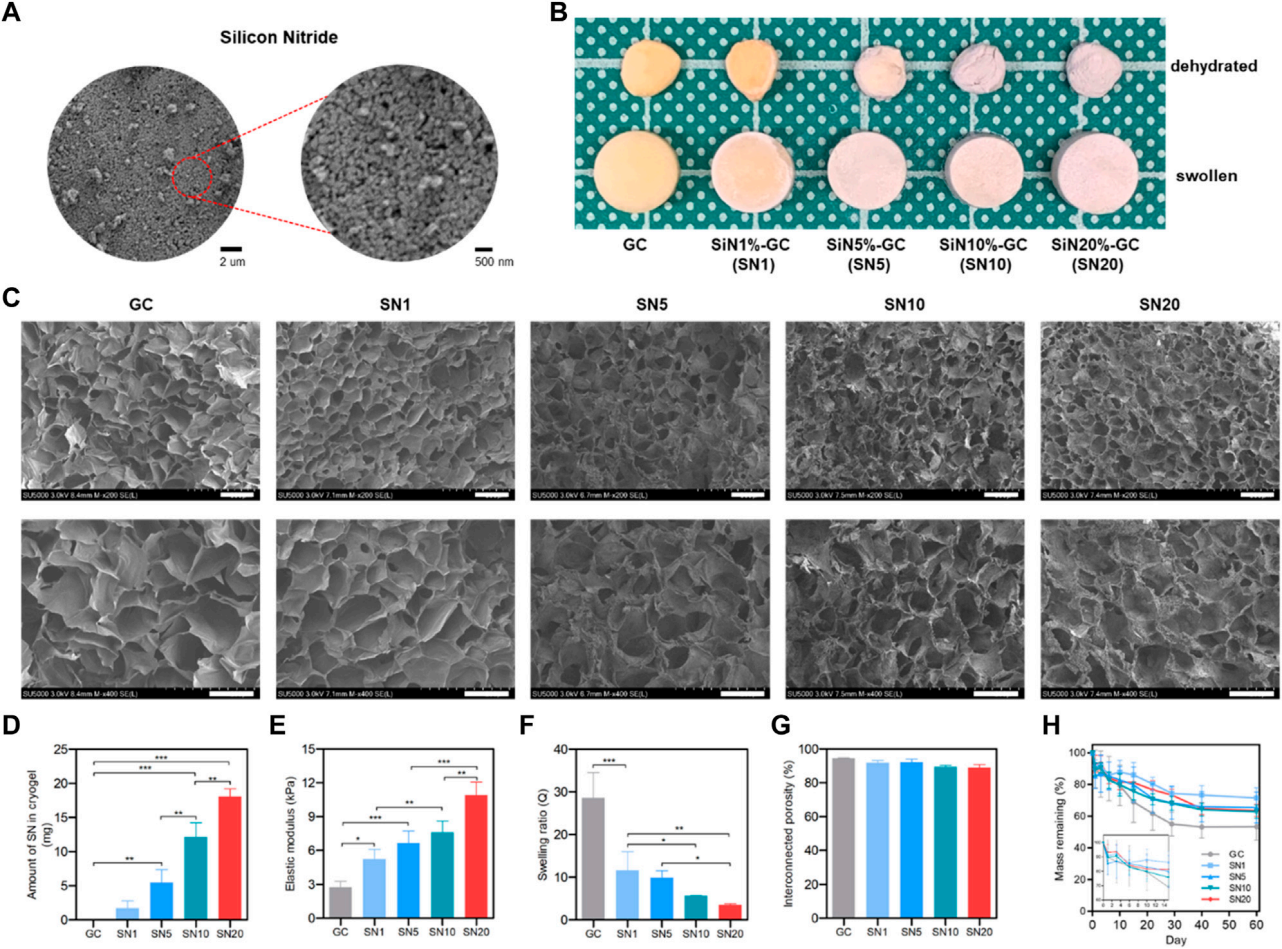
Characterization of SiN-GC cryogels. **(A)** Representative field emission scanning electron microscopy (FE-SEM) image of silicon nitride microparticles. **(B)** Representative photographs of gelatin/chitosan cryogel (GC) and SiN-GC with different concentrations of SiN (SN1, SN5, SN10, and SN20). The image shows the dehydrated and swollen states of GC and SiN-GCs (diameter of scaffold: 8 mm). **(C)** Representative FE-SEM images of SiN-GC cryogels. The images represent the cross sections of each scaffold. Scale bar of top and bottom images = 150 and 100 μm, respectively. **(D)** SiN loading efficiency into cryogel. After SiN-GCs were digested in papain solution, the weight of SiN remaining in cryogels was measured (*n* = 3). **(E)** Elastic modulus of SiN-GC cryogels from compression tests (*n* = 3). **(F)** Swelling ratio and **(G)** interconnected porosity of SiN-GC cryogels (*n* = 5). **(H)** Degradation rate of SiN-GC in 0.25% trypsin—EDTA solution (*n* = 5). Error bars indicate SD.

Lyophilizing ice crystals that were formed during the glutaraldehyde-mediated imine bond cross-linking between gelatin and chitosan at −20°C resulted in a macroporous structure of the gelatin/chitosan (GC) cryogel with sponge-like properties and biocompatibility. Prior to reinforcement of silicon nitride (SiN), SiN microparticles were analyzed with SEM and showed acicular polycrystal shape with a homogenous size of approximately 300 nm (Figure 2A). Four types of SiN-GC cryogels were prepared by loading different concentrations of SiN microparticles on GC cryogels (SN1: 1% of SiN, SN5: 5% of SiN, SN10: 10% of SiN, and SN20: 20% of SiN) to determine the concentration of SiN with the best efficiency. As shown in Figure 2B, we fabricated white cylindrical-shaped scaffolds and observed that the color of the scaffold became whiter as the SiN concentration in the cryogel increased, which shows successful SiN microparticle reinforcement in SiN-GC groups. In addition, the sizes of all cryogels in the fully swollen state or dehydrated state were similar, which indicated that SiN microparticles did not affect the morphology of the cryogel, e.g., sponge-like characteristics.

### Characterization of SiN-GC Cryogels

SEM of lyophilized cryogels was used to evaluate the microstructure of the scaffolds’ cross sections. All groups showed a uniform and porous structure with well-interconnected pores, indicating a homogeneous network constructed by a slow cross-linking reaction at subzero temperature (Figure 2C). For the SiN-GC groups, the SiN microparticles were homogenously distributed on the cryogels. In the case of SN10 and SN20, SiN microparticles were more dispersed and consistently coated the surfaces of cryogels, compared to the rest of the groups. Moreover, more SiN microparticles were observed on the SiN-GC groups with higher SiN concentrations. Regarding the structures of scaffolds, there was no significant difference in pore sizes or microstructures based on the observation of SEM images, suggesting a consistent macroporous structure in all cryogel groups.

From loading efficiency measurements, the highest amount of SiN was found embedded in SN20 (18.04 ± 1.16 mg), followed by SN10, SN5, and SN1 with 12.17 ± 2.07 mg, 5.47 ± 1.90 mg, and 1.71 ± 1.07 mg, respectively (Figure 2D). This confirms that SiN microparticles were successfully reinforced into GC cryogels except for the SN1 group which showed no statistically significant difference from the GC group. After confirming the SiN loading efficiency, we further determined the mechanical reinforcement with SiN. The Young modulus of SiN-GC groups increased significantly as the concentration of SiN increased (SN1 = 5.24 ± 0.86 kPa, SN5 = 6.65 ± 1.08 kPa, SN10 = 7.59 ± 1.01 kPa, and SN20 = 10.90 ± 1.16 kPa) compared to that of GC cryogel (2.74 ± 0.52 kPa) (Figure 2E). SiN reinforcement thus increased the mechanical stiffness of the scaffold.

We further measured the swelling ratio and interconnected porosity of GC and SiN-GC groups. As shown in Figure 2F, the GC scaffold showed the highest swelling ratio of 28.6 ± 5.9, which was significantly higher than that of SN1, SN5, SN10, and SN20 (swelling ratio of 11.6 ± 4.4, 9.9 ± 1.6, 5.6 ± 0.1, and 3.5 ± 0.3, respectively) due to increased weight of SiN compared to that of GC cryogels, which would, in turn, affect the total weight and swelling ratio calculation. Despite the difference in swelling ratio between the GC cryogel and SiN-GC groups, the interconnected porosity of all groups was above 85%, indicating that the pores of all scaffolds were well-interconnected (Figure 2G). This suggests that the SiN microparticles did not cause any obstructions between the pores of the scaffold, which would otherwise impede cell migration and bone ingrowth into the scaffold.

We additionally investigated the stability of the SiN-GC cryogels against enzymatic degradation. After 7 days of trypsin–EDTA incubation, all groups showed a similar degradation rate, which was ca. 20% (Figure 2H). However, after 10 days, the SiN-GC groups showed slower degradation than GC due to the higher density of SiN than gelatin and chitosan, which caused less reduction of mass in the remaining portion of the scaffold than in the group without SiN. After 60 days of incubation, GC and SN20 showed around 46.5 ± 8.5% and 36.3 ± 3.9% of degradation, respectively. However, there was no significant difference among the groups, which indicates that SiN reinforcement does not affect the degradation rate of the scaffold significantly, regardless of how much SiN was loaded.

Based on the characterization results, we excluded the SN1 group from further study, as it showed no significant SiN loading, compared to GC, and therefore proceeded further with the four groups (GC, SN5, SN10, and SN20) to investigate and compare more distinctive biological effects caused by different levels of SiN loading between the scaffold groups.

### Bioactivity of SiN-GC Cryogels

**FIGURE 3:**
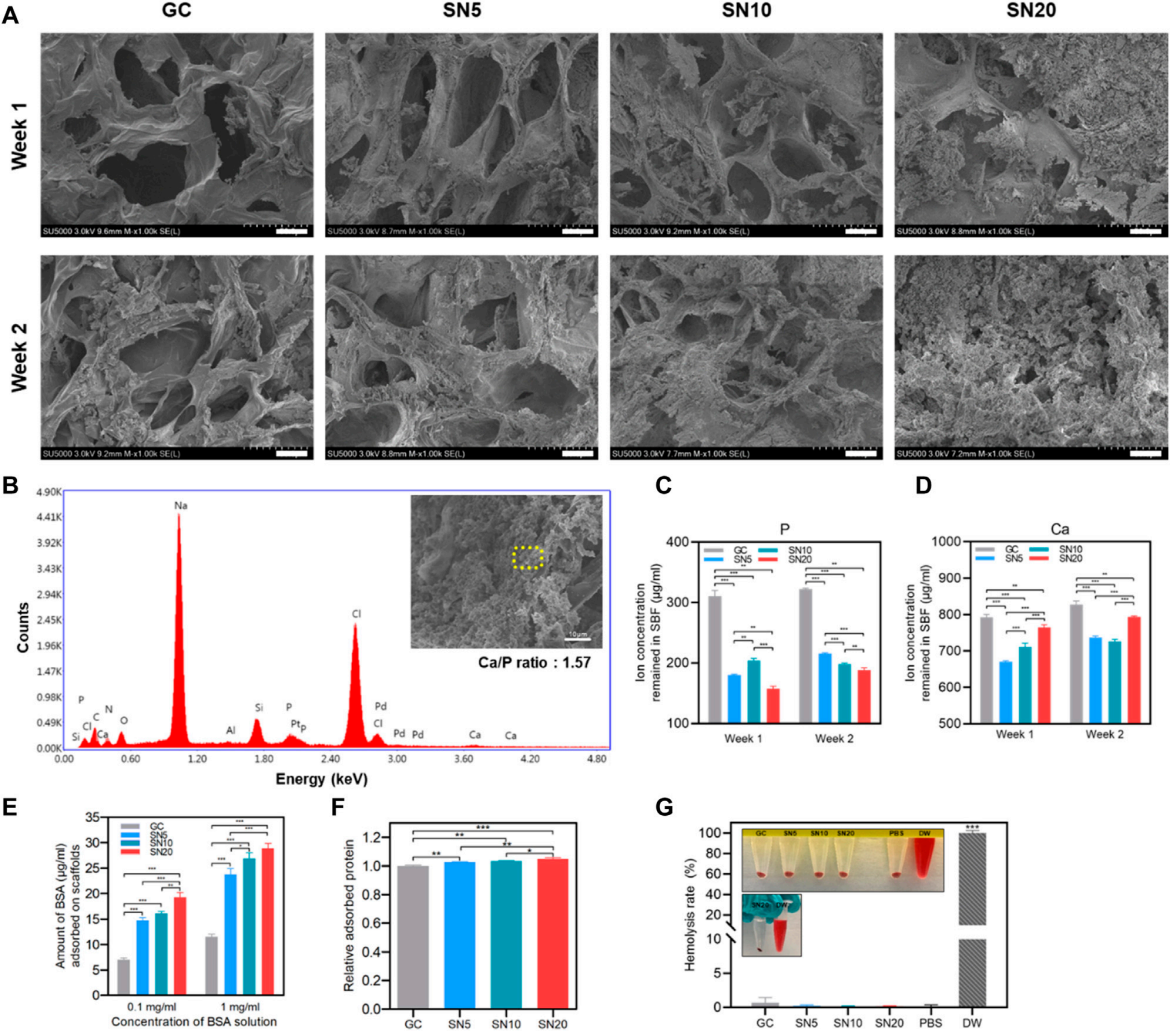
Apatite-forming and protein adsorption capacity of SiN-GC cryogels. **(A)** Representative FE-SEM images of GC, SN5, SN10, and SN20 after immersing in SBF at 37°C for 1 week and 2 weeks. The images show the cross sections of each scaffold (scale bar = 25 μm). **(B)** EDS spot analysis of an apatite particle that was formed in SN20 cryogels. The yellow dashed area shows the region of the EDS spot that was analyzed. EDS analysis confirms the existence of calcium and phosphate ions in the particle. **(C–D)** Quantified **(C)** phosphorous ion and **(D)** calcium ion concentrations remained in SBF 1 week and 2 weeks after immersion of scaffold. **(E)** Adsorbed bovine serum albumin (BSA) protein amounts on the testing groups, depending on 0.1 mg/ml and 1 mg/ml of BSA solution. The scaffolds were submerged in the BSA solution for 1 h. **(F)** Relative adsorbed protein on the scaffolds when submerged in growth medium with 10% fetal bovine serum. **(G)** Comparison of the hemolysis rate of GC, SN5, SN10, and SN20 with PBS (negative control) and DW (positive control). Error bars indicate SD (*n* = 3).

We further compared the bioactivity of the SiN-GC cryogels by investigating apatite formation on the scaffold under a simulated physiological environment. All the sample groups demonstrated apatite formation already after 1 week of incubation. However, there was a clear difference in the amount of apatites between the GC and SiN-GC groups (Figure 3A). While the GC cryogel could only promote small apatite particle formation on the surface of the cryogel, SN5, SN10, and SN20 cryogels all promoted significantly larger amounts of apatite deposition. The difference in apatite formation became even more apparent after 2 weeks of incubation. The surfaces of SN10 and SN20 scaffolds were almost completely covered with apatite particles (Figure 3A). In order to verify whether apatites were derived from calcium phosphate (CaP), energy-dispersive X-ray spectrometry (EDS) elemental mapping was performed to analyze the composition of the particles. As shown in Figure 3B, EDS analysis confirmed calcium, oxygen, and phosphorous ions in the precipitations, indicating that the formed particles were indeed CaPs. The Ca/P ratio obtained from the EDS analysis was 1.57, essentially in the range of CaPs between tricalcium phosphate (TCP, 1.5) and hydroxyapatite (HAP, 1.67). In order to compare the ion concentrations in the cryogels, we additionally collected the remaining SBF from each group and performed an inductively coupled plasma spectroscopy (ICP) analysis of the solution. At both week 1 and week 2, the GC group showed significantly higher remaining phosphorous and calcium ion concentrations than all SN5, SN10, and SN20 groups, which suggests that higher ion concentrations were used for apatite formation on the surfaces of SiN-GC groups (Figures 3C,D). This is due to the strong negative charges of SiN which causes calcium ion binding, followed by phosphorus ions to form more apatites than GC. In addition, acicular polycrystal microstructures of SiN increased the overall surface area of the scaffold which provides more area for apatite deposition. However, it should be noted that other factors such as pore size, hydrophilicity, and water evaporation during the experiment could have affected these results. To further test our hypothesis, calcium deposition of the scaffold was confirmed as well with Alizarin red S (ARS) staining in a later experiment.

Then, we examined the protein adsorption capacity of scaffolds by submerging them in bovine serum albumin (BSA) solution to determine which scaffold provides a favorable cell environment with protein binding. The amount of BSA adsorbed on SiN-GC groups was significantly higher than that in the GC group in both solutions (Figure 3E). Also, the increase in SiN concentration led to significantly higher protein adsorption capacity of SiN-GC. Although the difference of adsorbed BSA amount between SN5 and SN10 was not significant in 0.1 mg/ml BSA solution, SN20 demonstrated a significantly higher protein adsorption than the rest of the groups. Specifically, the protein adsorption capacity of SN20 was about three times that of GC. This suggests that the acicular polycrystal microstructures of hydrophilic SiN increased the surface area to adsorb more proteins than the control group. Furthermore, we performed another protein adsorption test with growth medium containing 10% fetal bovine serum (FBS) to mimic the *in vivo* environment. As shown in Figure 3F, although the adsorbed protein amount between groups was not as dramatically different as the 0.1 mg/ml or 1 mg/ml BSA solution experiment, it demonstrated similar results, i.e., all SiN-GC groups showed a significantly higher protein adsorption than GC, and higher SiN concentration in the scaffold led to a higher protein adsorption.

Along with bioactivity, the blood compatibility was also evaluated by the hemolysis rate measurement. The release of hemoglobin was analyzed, and DW and PBS treated with red blood cells were used as positive and negative controls, respectively. As shown in Figure 3G, the hemolysis rates of all SiN-GC groups were less than 0.7%. This indicates all SN5, SN10, and SN20 scaffolds do not cause any disturbance to red blood cells or hemolytic anemia, implying that all SiN-GC scaffolds have safe blood compatibility properties.

### Antibiofilm Activity and Inhibition of Bacterial Attachment of SiN-GC Cryogels

**FIGURE 4:**
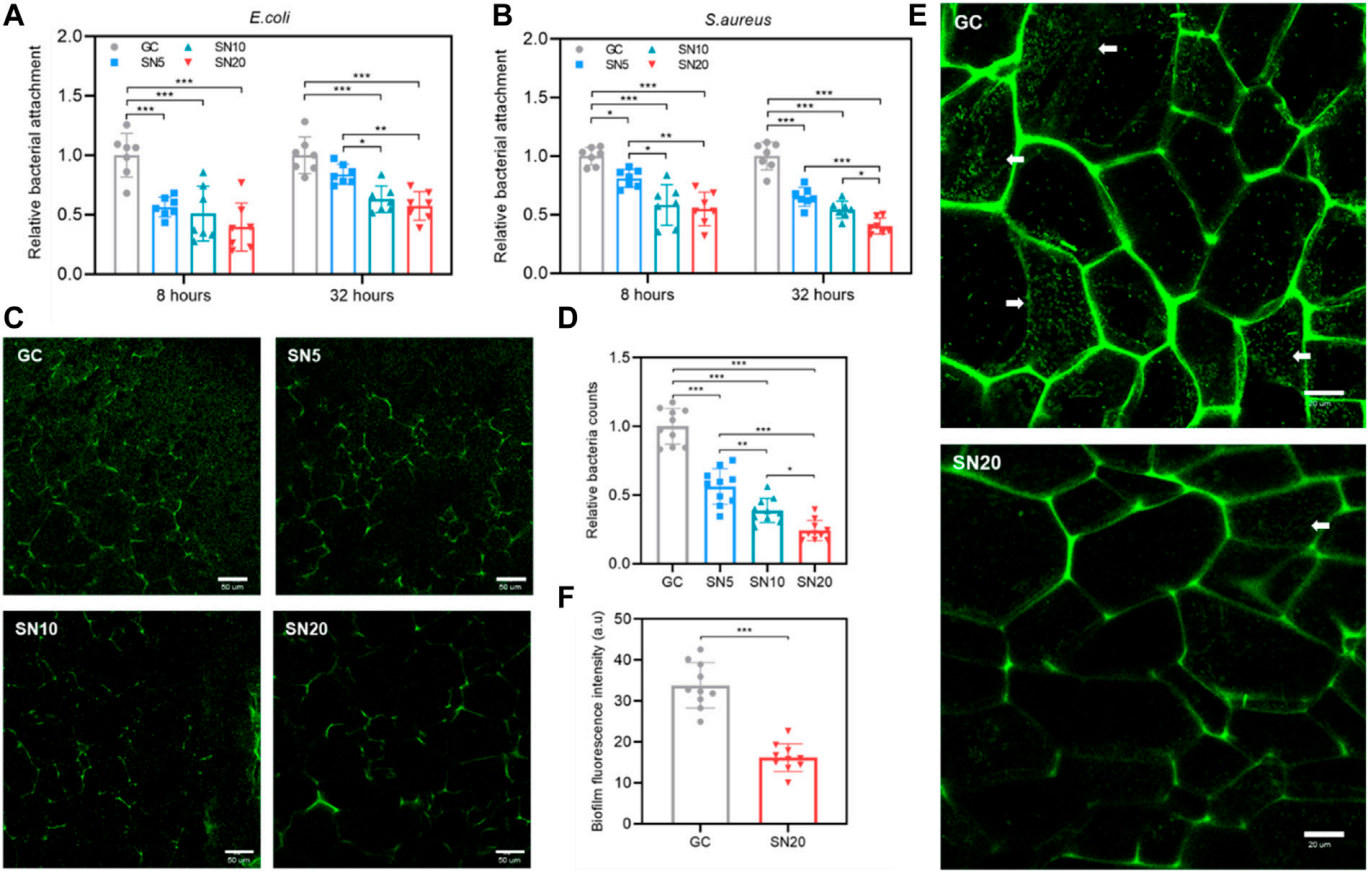
Antibiofilm effect and inhibition of bacterial attachment of SiN-GC cryogels. **(A–B)** Quantitative analysis of bacterial attachment of **(A)** *E. coli* and **(B)** *S. aureus* in GC, SN5, SN10, and SN20 cryogels after 8 and 32 h of culture. Attached bacteria were quantified by the Presto blue assay (*n* = 7). **(C–D) (C)** Representative confocal laser scanning microscopy (CLSM) images and **(D)** quantified relative bacteria counts showing the attachment of *E. coli* expressing green fluorescent protein (GFP) (GFP-*E.coli*) to the testing groups after 32 h of culture. **(E–F)** Biofilm inhibiting capacity of SiN-GC cryogels. **(E)** Representative CLSM images of inner cross sections of GC and SN20 cryogels that were seeded with GFP-*E. coli* and cultured for 32 h. White arrow represents the biofilm formed by GFP-*E. coli*. **(F)** Quantified analysis of relative fluorescence intensity of the biofilm formed in GC and SN20. Error bars indicate SD (*n* = 10).

Antibiofilm and antibacterial properties of SiN-GC cryogels were evaluated by incubating the scaffolds in growing *E. coli* and *S. aureus* static cultures. For *E. coli*, the SN20 group showed the lowest bacterial attachment among the group at both 8 and 32 h (0.40 ± 0.20 and 0.57 ± 0.12-fold lower than GC at 8 and 32 h, respectively) (Figure 4A). As shown in Figure 4B, for *S. aureus*, a similar result was obtained that all SN5, SN10, and SN20 groups demonstrated significantly lower bacterial attachment than the GC group (0.81 ± 0.08-, 0.58 ± 0.17-, and 0.55 ± 0.14-fold lower at 8 h and 0.65 ± 0.08, 0.54 ± 0.07, and 0.40 ± 0.07 at 32 h, respectively). In addition, we confirmed again that cryogels with higher SiN concentrations resulted in less bacterial attachment. The SN20 group showed the highest antibiofilm activity, reducing the attachment by approx. 50% compared to the control group. We checked the absorbance of planktonic culture after the experiment to see if SiN-GC had a long-term bactericidal effect. However, there was no statistically significant difference between groups, and SiN-GC groups generally exhibited a lower absorbance value than the GC group in both the *E. coli* and *S. aureus* experiments (Supplementary Figures S1A,B).

We also used *E. coli* expressing green fluorescent protein (GFP) (GFP-*E. coli*) to visualize the antibiofilm effect of SiN-GC using confocal laser scanning microscopy (CLSM). As shown in Figure 4C, significantly less GFP-*E. coli* cells were attached to the SiN-GC scaffolds compared to the GC after 32 h of incubation. To verify this result quantitatively, we used the CLSM software to count the bacteria in the sample images, and the relative counts of attached bacteria on GC, SN5, SN10, and SN20 surfaces were 1.00 ± 0.13, 0.56 ± 0.14, 0.38 ± 0.09, and 0.24 ± 0.08, respectively (Figure 4D). This confirms our previous results showing that the SiN-GC groups with higher SiN showed a significantly stronger antibiofilm effect. We further evaluated the cross section of the scaffolds using CLSM and performed a direct comparison of antibiofilm property between GC and SN20. Easily distinguishable biofilms with web-like morphology were formed by GFP-*E. coli* cells on the GC scaffold, which was not the case for SN20 (Figure 4E). Then, to confirm the antibiofilm activity of GC and SN20 with quantitative data, the biofilm fluorescence intensity of randomly selected biofilms in each group was analyzed. As shown in Figure 4F, the fluorescence intensities of biofilm in GC and SN20 groups were 33.8 ± 5.7 and 16.2 ± 3.4, respectively. Taken together, our data strongly suggest an effective inhibition of bacterial attachment and antibiofilm activity and of SN20.

### Cellular Activity and Proliferation of SiN-GC Cryogels

Before evaluation of cellular activity on SiN-GC cryogel, first we checked the cellular response of MC3T3-E1 on 2D culture using general media with SiN 0% (control), 1, 2, 3% (w/v) concentration to check biocompatibility and proliferation. As shown in Supplementary Figures S2A,B, all groups showed greater than 98% viability, confirming the biocompatibility of SiN microparticles for use in a biomaterial scaffold. For the proliferation rate, although there was no statistically significant difference, the result showed a tendency toward a higher cell proliferation rate as the SN concentration in media increased (Supplementary Figure S2C).

**FIGURE 5:**
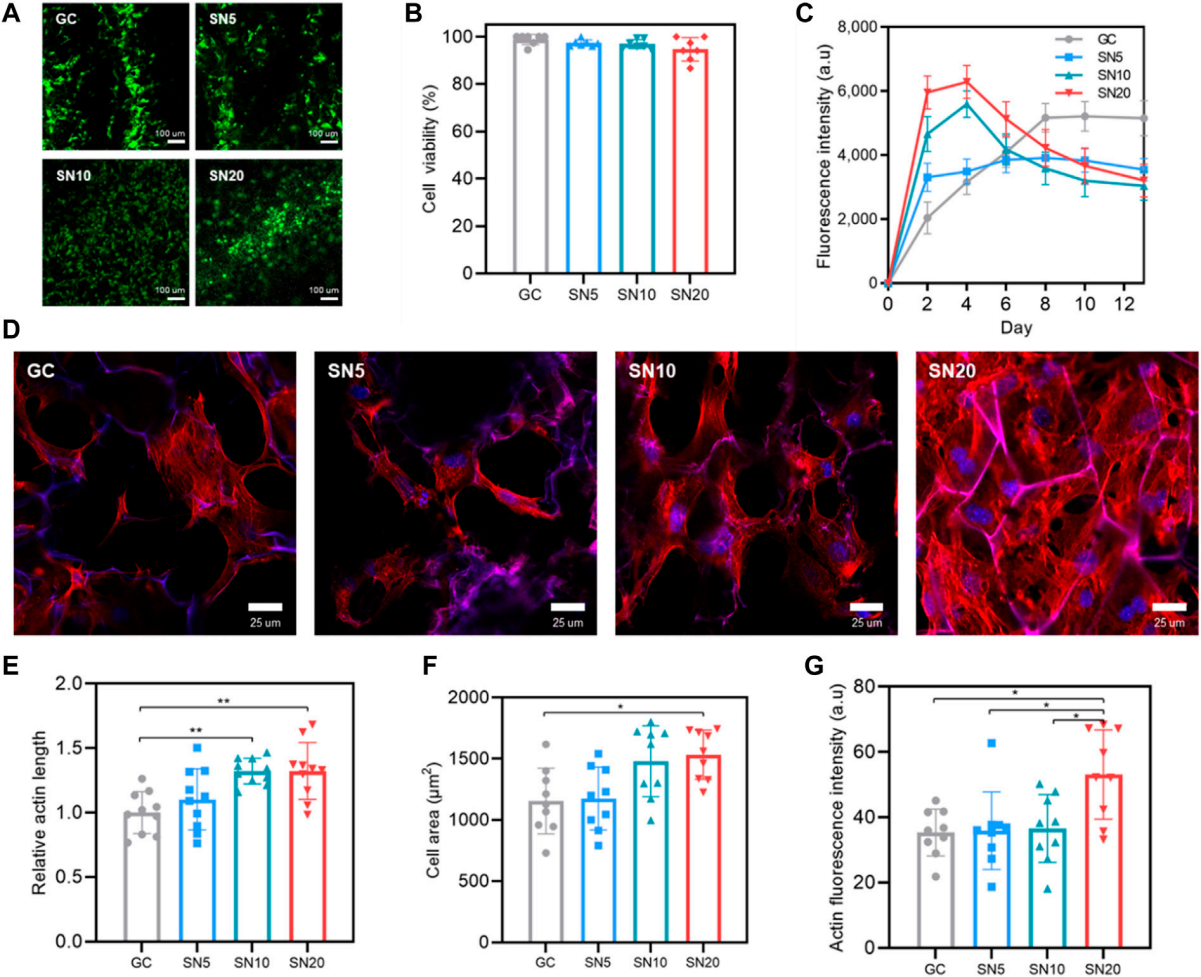
Biocompatibility, proliferation, and cell morphology in SiN-GC cryogels. **(A)** Representative CLSM images show the Live/Dead fluorescent assay of MC3T3-E1 pre-osteoblasts that have been seeded on GC, SN5, SN10, and SN20 (green: live, red: dead). 5 × 10^4^ cells were seeded on scaffolds and stained by the Live/Dead assay after 24 h of seeding. **(B)** Quantitative analysis of cell viability from the Live/Dead assay. **(C)** Proliferation rate of pre-osteoblast cells that were seeded on GC, SN5, SN10, and SN20. 5 × 10^3^ cells were seeded on scaffolds, and cell proliferation was measured by the Presto blue assay (*n* = 4). **(d)** Representative CLSM images that show the staining of actin microfilament cytoskeletal protein (red) and nuclei counterstained with DAPI (blue) of the cells after 3 days of culturing on GC, SN5, SN10, and SN20 cryogels. **(E–G)** Quantitative analysis of **(E)** relative actin length, **(F)** cell area, and **(G)** actin fluorescence intensity in response to GC, SN5, SN10, and SN20 cryogels based on the CLSM images of actin/DAPI stained cells. Data were measured and analyzed by CLSM software based on randomly chosen cells in three independent experiments (*n* = 10). Error bars indicate SD.

Based on 2D *in vitro* results, we moved on to investigate cellular activity on the SiN-GC cryogels. For cell viability, the CLSM images show that most of the cells in all groups were alive (Figure 5A). In addition, based on quantified results from the Live/Dead assay, GC, SN5, SN10, and SN20 all exhibited cell viability above 94%, which indicates all scaffolds are biocompatible even if the SiN concentration in the scaffold increases (Figure 5B). For the proliferation rate, until day 6, SN20 and SN10 exhibited the highest and the second-highest cell proliferation rate, while GC showed the lowest cell proliferation rate (Figure 5C). On day 2, SN20 showed about a threefold higher cell proliferation rate than GC. However, after day 6, while the GC group showed an increase in the proliferation rate, the cell proliferation of all SiN-GC groups started to decline, due to the SiN enabling the cells in the SiN-GC groups to reach a plateau faster than the GC group, and its osteoconductivity inducing osteogenic differentiation.

Furthermore, cells on the scaffolds were stained with phalloidin and DAPI to analyze cellular morphology. Most of the cells in all groups showed a round and well-spread morphology on the scaffold (Figure 5D). However, there were some differences between the GC group and SiN-GC groups that while some cells in the GC group showed an elongated spindle-shaped morphology without stress fibers, the cells in SiN-GC groups especially in SN20 exhibited a round polygonal cellular morphology with distinct and thick stress fibers. However, since the differences among SN5, SN10, and SN20 were marginal, we used CLSM software to quantify the actin length, cell area, and actin fluorescence intensity of the cells in each group. For actin length, while there was a statistical difference in SiN-GC groups, SN10 and SN20 showed significantly longer actin length than GC (Figure 5E). Furthermore, SN20 was the only group with a significantly higher cell area and actin fluorescence intensity than the GC, suggesting stronger focal adhesion and better biocompatibility (Figures 5F,G). Our data thus confirm the previous results on cell proliferation and quantified cell morphology.

### Mineralization and Osteogenic Effect of SiN-GC Cryogels

Following cellular activity, the mineralization and osteogenic effect of SiN-GC cryogels were evaluated. First, ALP and ARS staining was used to analyze osteoconductivity of SiN via 2D *in vitro* cell culture with SiN-conditioned medium (OM). The SN3% group showed the highest level of osteogenesis with the highest ALP activity, while the rest of the groups, except for the control group, showed moderate osteogenesis (Supplementary Figure S3A). Similarly, ARS staining on day 7 showed that SN3% reached the highest level of calcification (Supplementary Figure S3B). Quantification confirmed that SN3% showed a significantly higher osteogenic differentiation than the rest of the groups at both time points (Supplementary Figure S3D). Additionally, the same result for ARS was demonstrated on day 14, as well as a significantly higher level of calcification for the SN3% group (Supplementary Figures S3C,E). Interestingly, the SN1% group showed about the same level of osteogenic differentiation compared to the control group, suggesting that a certain level of SN is required for enhanced osteogenic differentiation.

**FIGURE 6:**
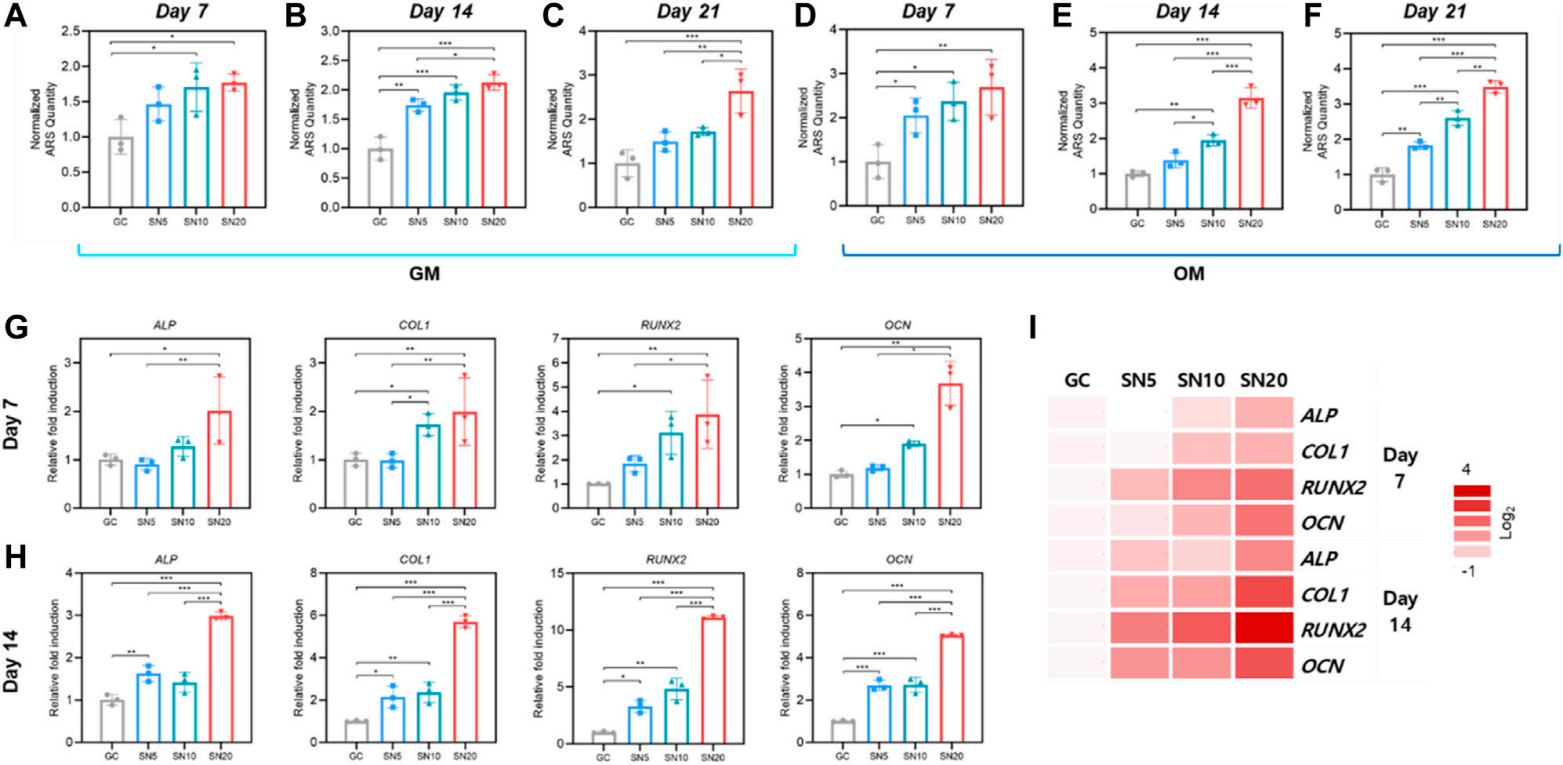
Mineralization and osteogenic effect of SiN-GC cryogels. **(A–F)** Quantitative analysis of mineralization by Alizarin red S (ARS) staining after **(A,D)** 7 days, **(B,E)** 14 days, and **(C,F)** 21 days of culturing pre-osteoblasts with **(A–C)** general media (GM) or **(D–F)** osteogenic media (OM) on GC, SN5, SN10, and SN20. **(G–H)** Relative fold induction of osteogenic genes on **(G)** day 7 and **(H)** day 14. Pre-osteoblasts were seeded on the scaffolds and cultured with osteogenic medium. **(I)** Heatmap of osteogenic gene profiles from RT-qPCR after 7 and 14 days of osteogenic differentiation (red = upregulations, white = downregulations). Error bars indicate SD (*n* = 3).

In 3D cell-laden SiN-GC cryogels, SN10 and SN20 demonstrated significantly higher calcium deposition on day 7 than GC, and the group with higher SiN concentration exhibited higher mineralization (Figure 6A). On day 14 and day 21, the ARS staining presented similar results as the data on day 7 (Figures 6B,C). In addition, although there was no significant difference between SN5 and SN10, SN20 showed the highest level of calcium deposition among the groups. On day 21, the mineralization from SN20 was significantly higher than that of GC, SN5, and SN10. Furthermore, we repeated ARS staining to investigate mineralization under osteogenic medium (OM) conditions to determine whether SiN can bring a synergistic effect in osteogenesis with osteogenic factors in the media. As Figure 6D shows, unlike previous experiments under GM conditions, SN5, SN10, and SN20 showed significantly higher mineralization than the GC group on day 7. Similarly, on days 14 and 21, the SiN-GC group with higher SiN yielded higher calcium deposition, and SN20 showed the highest calcium deposition than the rest of the group (Figures 6E,F). In general, we were able to confirm that the overall mineralization difference between GC and SiN-GC groups under culturing conditions with OM was greater than that in the experiment under culturing conditions with GM. This indicates that osteoconductivity of SiN from SiN-GC was able to exhibit a synergistic effect on osteogenesis, and the higher concentration of SiN further enhances the osteogenic effect.

We further focused on osteogenic gene expression of pre-osteoblasts by quantitative real-time PCR (RT-qPCR) after seeding on the scaffolds and culturing with OM for 7 and 14 days. In line with ARS analysis, on day 7, the SN20 group exhibited the highest expression of osteogenic markers *ALP*, *COL1*, *RUNX2*, and *OCN*, with 2.0 ± 0.69, 2.0 ± 0.69, 3.9 ± 1.4, and 3.7 ± 0.64-fold higher than those of GC, respectively (Figure 6G). The SN10 group showed the second-highest osteogenic gene expressions and significantly higher gene expression of *COL1*, *RUNX2*, and *OCN* than GC. However, in case of SN5, the osteogenic gene expressions of SN5 showed a slightly upregulated osteogenic gene expression than GC; however, the difference was insignificant. On day 14, the gene expression from RT-qPCR demonstrated a similar result as that of day 7. As shown in Figure 6H, the SN20 group exhibited the highest expression of osteogenic markers *ALP*, *COL1*, *RUNX2*, and *OCN*, 3.0 ± 0.09, 5.7 ± 0.29, 11.1 ± 0.13, and 5.1 ± 0.04-fold, respectively, higher than GC and the rest of the groups. SN10 showed significantly higher gene expression in *COL1*, *RUNX2*, and *OCN* than GC. Moreover, the osteogenic gene expression of SN5 was significantly upregulated compared to GC. Overall, as the heatmap of osteogenic gene profiles from all groups shows, we noted a significant upregulation of osteogenic markers in scaffolds with higher SiN concentration and an increase in gene expression difference over time between SiN-GC groups and the GC group (Figure 6I).

### Osteogenic Profile of SiN-GC Cryogels Under Cyclic Loading in the Bioreactor

**FIGURE 7:**
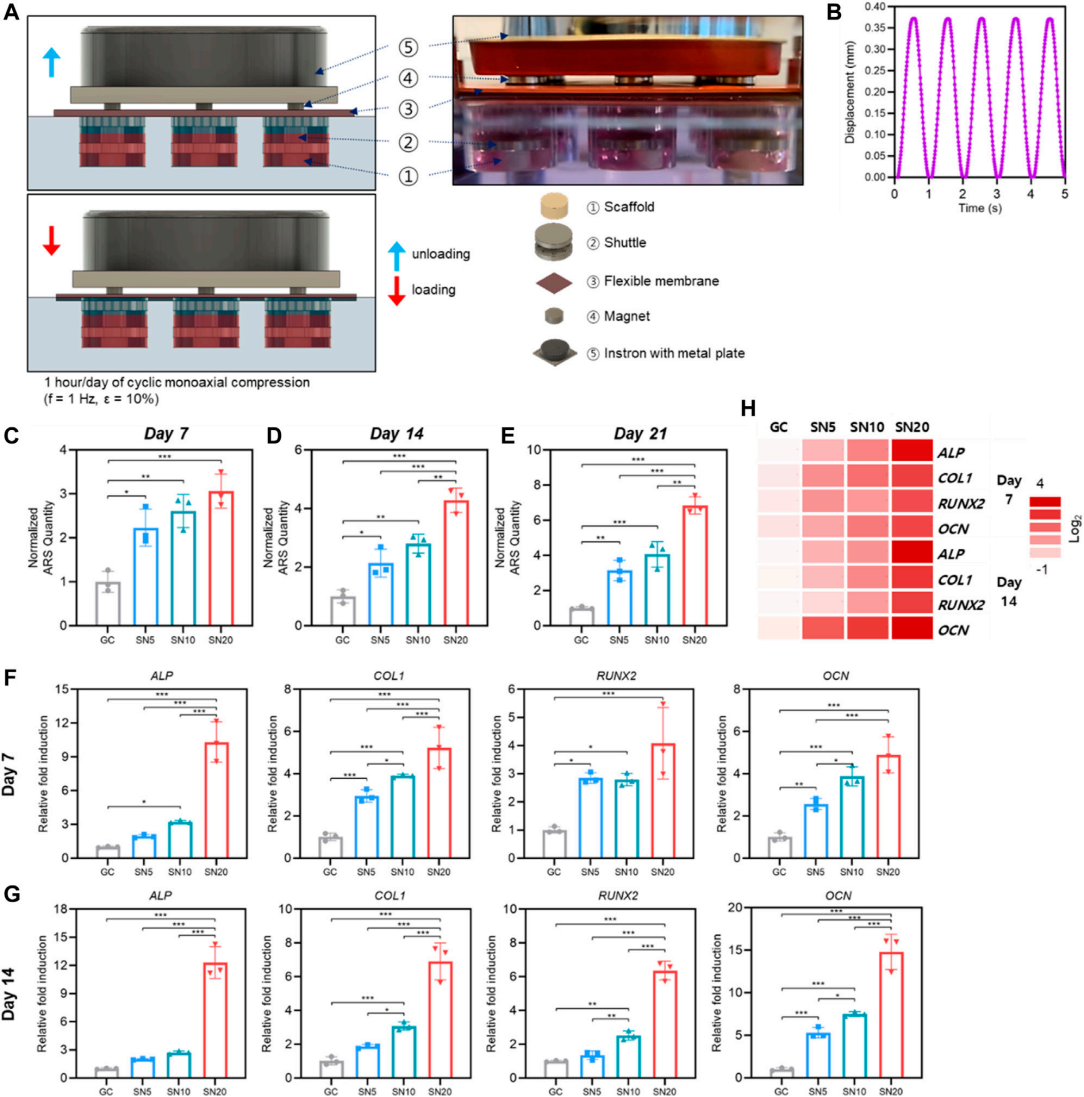
Osteogenic profile of SiN-GC under cyclic loading by a bioreactor. **(A)** Schematic illustration with a side view photograph shows the cyclic loading mechanism of the bioreactor. **(B)** Waveform graph that shows the cyclic loading condition used in this study (1 h/day of cyclic compression, f = 1 Hz and *ε* = 10%). **(C–E)** Quantitative analysis of mineralization by Alizarin red S (ARS) staining after **(C)** 7 days, **(D)** 14 days, and **(E)** 21 days of culturing pre-osteoblasts with osteogenic media (OM) on GC, SN5, SN10, and SN20 under cyclic loading by a bioreactor. **(F–G)** Relative fold induction of osteogenic genes on **(F)** day 7 and **(G)** day 14. Pre-osteoblasts were seeded on the scaffolds and cultured with osteogenic medium under cyclic loading by a bioreactor. **(H)** Heatmap of osteogenic gene profiles from RT-qPCR after 7 and 14 days of osteogenic differentiation under cyclic loading condition (red = upregulations, white = downregulations). Error bars indicate SD (*n* = 3).

Then, we evaluate the osteogenic profile of SiN-GC cryogels by 3D *in vitro* culture under simulated physiological cyclic loading conditions in a custom-made mechanobioreactor system (Figure 7A; Supplementary Figure S4). The loading condition was set to be 1 h/day of cyclic monoaxial compression with a frequency of 1 Hz and strain of 10% as the waveform graph shown in Figure 7B, and all the scaffolds did not show any deformation during or after the cyclic loading due to sponge-like characteristics of GC cryogel (Supplementary Video S1).

Starting from day 7, ARS staining showed that all SiN-GC groups (SN5, SN10, and SN20) demonstrated significantly higher mineralization than the GC group (Figure 7C). Similar to ARS staining under static conditions, the group with higher SiN concentration showed more calcium deposition. For SN5 and SN10, both groups showed significantly higher mineralization than the GC group on days 14 and 21, and the mineralization of SN10 was higher than that of SN5, but the difference was not significant (Figures 7D,E). For SN20, calcium deposition was highest among the groups, and the mineralization of SN20 was significantly higher than the mineralization of the other groups on days 14 and 21. In addition, interestingly, overall ARS results demonstrate that scaffolds cultured under cyclic loading conditions resulted in higher mineralization than the scaffolds cultured under static conditions.

In line with RT-qPCR results from static conditions, the group with higher SiN showed significant upregulation of osteogenic gene expression. On day 7, osteogenic genes in the SiN-GC groups were upregulated compared to GC (Figure 7F). Specifically, SN10 and SN20 showed a significantly higher expression of osteogenic markers *ALP*, *COL1*, *RUNX2*, and *OCN*, with 3.2 ± 0.12, 3.9 ± 0.08, 2.8 ± 0.22, and 3.9 ± 0.45-fold (SN10) and with 10.3 ± 1.79, 5.2 ± 0.98, 4.1 ± 1.3, and 4.90 ± 0.85-fold (SN20) upregulation compared to those of GC, respectively. In contrast to insignificant changes in osteogenic gene expression under static conditions, insignificant expression of *COL1*, *RUNX2*, and *OCN* under cyclic loading conditions was significantly higher in SN5 than GC. On day 14, RT-qPCR results showed similar trends, but the difference in gene expression between SiN-GC groups and the GC group was greater, especially for the SN20 group. As shown in Figure 7G, SN20 demonstrated the highest expression of osteogenic markers *ALP*, *COL1*, *RUNX2*, and *OCN*, with 12.3 ± 1.70, 6.9 ± 0.90, 6.4 ± 0.45, and 14.8 ± 1.69-fold higher expression than in the GC group and the rest of the groups. In addition, in line with previous experiments, the scaffolds with higher SiN concentration led to higher osteogenic gene expression, which shows the relationship between osteogenic effect and SiN.

Based on the results, we analyzed and compared the osteogenic profile between cyclic loading conditions by bioreactor and static conditions. As shown in Supplementary Figure S5, the expression level of osteogenic genes under cyclic loading conditions in a bioreactor is generally higher than that under static conditions in all scaffold groups. Besides, although the difference was not significant in all testing genes, it was clearly shown that SN20 led to higher osteogenic gene upregulation in bioreactor compared to static condition than GC did in bioreactor compared to static condition which indicates the osteogenic effect of SiN. Overall, the heatmap of osteogenic gene profiles under cyclic loading conditions shows that significant upregulations of osteogenic markers were found in the scaffold with higher SiN concentration, and the osteogenesis in SiN-GC groups get enhanced over time due to the synergistic effect from osteoconductivity of SiN microparticles and mechanotransduction during the dynamic loading in the bioreactor (Figure 7H).

## Discussion

In the present study, we developed a SiN-GC scaffold system composed of GC cryogel that is reinforced with SiN microparticles to achieve antibacterial and osteogenic effects with an enhanced cellular activity for optimal bone regeneration. We fabricated the GC cryogel by cryogelation with a glutaraldehyde-mediated cross-linking reaction to form an imine group between two amine groups from gelatin and chitosan (Bakhshpour et al., 2019; Lee et al., 2020). The GC component has two key advantages as a scaffold: cellular environment by biocompatibility and hydrophilicity and a macroporous structure. Gelatin and chitosan are biocompatible and hydrophilic materials that have been used widely in bone tissue engineering, and a cryogel ensures the hydrophilicity for the cellular environment (Di Martino et al., 2005; Doty et al., 2014; Amirthalingam et al., 2018; Kwon et al., 2018). In addition, the macroporous structures of the GC and SiN-GC cryogels caused by ice crystals during cryogelation are essential not only for cell infiltration but also for the efficient nutrient flow and the vascularization for tissue healing (Bencherif et al., 2012; Kim et al., 2018). The SiN component of the SiN-GC cryogel system is essential to provide enhanced mechanical support for the defect area and to incorporate antibiofilm and osteogenic properties in the scaffold (Pezzotti et al., 2017b; Pezzotti, 2019). From the SEM images and loading efficiency, we were able to confirm that SiN microparticles were incorporated successfully and homogenously coated on the surfaces of SN5, SN10, and SN20 (Figures 2C,D).

Based on the results of characterization, reinforcement of SiN in SiN-GC overcame the limitations of GC cryogel, such as low mechanical strength, yet still carried the unique advantages of GC such as hydrophilicity and interconnected porosity. SiN-GC scaffolds with higher SiN concentration demonstrated a higher elastic modulus under compressive loading (Figure 2E). A higher stiffness of SiN-GC scaffolds due to SiN incorporation is more likely to induce osteogenic differentiation than GC, which has relatively low mechanical stiffness, a known limitation of such hydrogels (Slaughter et al., 2009). The low mechanical stiffness of the scaffold is not favorable for osteogenic differentiation, since the stiffness of the scaffold affects the cell signaling and focal adhesions that would lead cells to differentiate into a tissue that has a similar stiffness to the scaffold (Engler et al., 2006; Bose et al., 2012; Oh et al., 2016; Lee et al., 2021b). Therefore, SiN reinforcement in SiN-GC increased the elastic modulus which would increase focal adhesions, cell proliferation, and osteogenic differentiation for bone tissue regeneration and enhanced mechanical support for the defect area (Figure 2E) (Nam et al., 2011; Chen et al., 2015). In addition, results from degradation experiments demonstrated that both GC and SiN-GC groups would provide a long-term stable mechanical support as a scaffold to ensure safe bone regeneration. At the same time, although SiN reinforcement increased the mechanical stiffness of the scaffold, the high interconnected porosity of SiN-GC was not affected by SiN (Figure 2G). This indicates that the incorporation of SiN did not cause any blockage in the pores of SiN-GC, and it can keep highly interconnected pores for cell migration and nutrition flow in the scaffold (Bose et al., 2012).

The bioactivity difference between GC and SiN-GC was likely facilitated by the negative charge and acicular polycrystal microstructure of SiN (Figure 3). First, the negative charge from the SiN surface attracts positively charged calcium ions from the surrounding environment (Leonor et al., 2007; Bock et al., 2017; Moskalewicz et al., 2018). The calcium ions are neutralized by binding with phosphate ions, which eventually leads to the apatite formation that was confirmed with EDS and ICP analysis (Figures 3B–D) (Leonor et al., 2007; Wu et al., 2020). Additionally, the increased surface area of SiN-GC due to the acicular polycrystal microstructure of SiN was another factor to induce more apatite deposition and the protein adsorption on the scaffold, which was also confirmed by other studies (Liu and Nemat-Nasser, 1998; Gould et al., 2009; Pezzotti et al., 2017a; Sainz et al., 2020). Consequently, SiN-GC scaffolds with higher SiN concentration result in higher biomineralization and protein adsorption, so SN20 exhibited the highest bioactivity among the groups (Figures 3E,F). This would be a valuable aspect *in vivo*, since the adsorbed proteins and formed CaP particles on the scaffold would not only enhance cellular activity but also provide an intrinsic osteogenic environment for cells to accelerate bone formation (Oyane et al., 2005; Yao et al., 2013; Sainz et al., 2020; Wu et al., 2020). Additionally, the hydrophilicity of the cryogel and SiN would provide favorable conditions for proteins to preserve a natural conformation (Peppas et al., 2006; Rabe et al., 2009; Slaughter et al., 2009; Bal and Rahaman, 2012; Awad et al., 2019).

Comparing the antibiofilm properties of SiN-GC scaffolds, we confirmed that the scaffold groups with higher SiN concentration resulted in a stronger inhibition of bacterial attachment and biofilm formation (Figure 4). One possible reason for this might be the nitric oxide (NO) that is produced from the surface of SiN when SiN is exposed to an aqueous solution (Pezzotti, 2019). NO is a short-lived gaseous molecule that can affect signaling and control fundamental metabolism in both prokaryotic and eukaryotic cells (Saura et al., 2010). However, above a certain concentration threshold, NO can be toxic by covalently binding with DNA, proteins, and lipids which lead to inhibition of the cells (Schairer et al., 2012; Pezzotti, 2019). In other words, NO diffuses across cellular membranes and induces nitrosative and oxidative damage, thereby compromising bacterial growth and survival. On the other hand, due to different NO concentration thresholds between cells and bacteria, cells on SiN do not suffer any side effects (Schairer et al., 2012; Seabra et al., 2016). This nitric oxide surface chemistry on SiN has been proven by other researchers. For instance, Pezzotti et al. used time-lapse fluorescent imaging to monitor NO formation in both mammalian and bacterial cells using a membrane permeable indicator and diaminofluorescein-2 diacetate [DAF-2(NO)] and confirmed an increase in NO concentration on the surface of SiN, which had an antibacterial effect on *S. epidermidis* but no negative effect on osteoblast cells at the same time (Pezzotti et al., 2017b; Pezzotti, 2019). These can explain the high cell viability and biocompatibility on all SiN-GC scaffolds from the Live/Dead assay (Figures 5A,B). In addition, since NO is short-lived and short-range, all of these bactericidal activities occurred very close to the surface of the SiN-GC, which may explain the lack of difference between groups in absorbance measurement from planktonic culture (Supplementary Figure S1).

Regarding the cellular activity on scaffolds, although all scaffold groups showed a similar degree of high cell viability, there were some differences in cell proliferation and morphology. Compared to the GC group, SiN-GC groups showed higher cell proliferation in early stages and reached a plateau sooner due to the osteoconductivity of SiN which induces cells to osteogenic differentiation (Figure 5C). Moreover, the morphology of the cells seeded on the scaffolds with higher SiN concentration tended to have more polygonal and round morphology with distinct stress fibers, which was also confirmed by quantitative analysis of phalloidin and DAPI staining (Figures 5D–G). One of the reasons for the enhanced cellular activity and morphology difference in SiN-GC groups might reside in the presence of nitrogen on the SiN surface. This nitrogen on the SiN surface induces the formation of N-H moieties that can act as precursors to the amide groups that are present in the extracellular matrix (ECM) (Awad et al., 2019). Another reason for enhanced cellular activity would be the increased surface area due to the acicular polycrystal microstructure of SiN which enables increased protein adsorption.

We further confirmed a higher calcium deposition of SiN-GC than that of GC under both conditions of culturing with GM or OM and higher calcium deposition from the scaffolds with higher SiN concentrations. Especially, the ARS results under GM culture conditions demonstrate that SiN-GC can induce a stronger mineralization than GC even without osteogenic factors in the media (Figures 6A–C). This indicates that the SiN-GC scaffold itself was able to induce enhanced cell proliferation, osteogenic differentiation, and mineralization by providing a macroporous and interconnected structure, osteoconductivity of SiN, and mechanically stronger microenvironment from SiN reinforcement (Murphy et al., 2010; Kim et al., 2018; Lee et al., 2020). Consequently, ARS results under culture conditions with OM demonstrated an even higher mineralization of SiN-GC groups than GC due to the synergistic osteogenic effect from OM and SiN-GC. Similar results were obtained from RT-qPCR, showing that the groups with higher SiN concentration exhibited a higher osteogenic gene expression, especially SN20 (Figures 6G,H). As previously mentioned, there are many possible factors that could lead to these results, but one of the main reasons for the strong osteogenic effect of SiN-GC is the osteoconductivity of SiN microparticles. The osteoconductivity of SiN has not been fully clarified yet. However, previous studies suggested that the surface chemistry of SiN has a vital role in stimulating osteoblast proliferation and induction of bone formation (Pezzotti et al., 2017a; Awad et al., 2019; Zanocco et al., 2019). Zanocco et al. investigated the role of silicon and nitrogen in SiN by evaluating osteogenic response from SiN substrate with modified surface stoichiometry (Zanocco et al., 2019). As the surface stoichiometry of SiN was gradually altered toward a silicon-rich composition, the cell proliferation and osteogenic response reduced with decreasing nitrogen content. Thus, nitrogen from SiN plays an important role in stimulating osteogenic response and has a synergistic effect in bone tissue formation, with silicon stimulating early stages of bone formation and calcification (Shie et al., 2011; Khan et al., 2014; Kwon et al., 2018). Additionally, NO from SiN not only shows antibacterial properties but also affects bone resorption by inducing apoptosis of osteoclasts, which would eventually lead to bone formation *in vivo* (Pezzotti, 2019; Lee et al., 2021a).

Finally, we evaluated whether SiN-GC would show a similar mineralization and osteogenic profile under simulated physiological conditions by culturing under cyclic compressive loading in a bioreactor (Figure 7). It has been reported that interfragmentary movement enhances fracture healing in multiple animal studies, and the interfragmentary compressive strain from weight-bearing in experimental *in vivo* fracture healing studies was reported to range between around 10 and 20% (Klein et al., 2003; Schell et al., 2008; Klosterhoff et al., 2017). In addition, according to the other studies investigating the impact of mechanical loading on cell differentiation, a strain in the range of 10% has the strongest influence on osteogenic differentiation (Jagodzinski et al., 2008; Rath et al., 2008; Li et al., 2010; Jeon et al., 2017). For this reason, we applied a strain of 10% in this study to investigate the fatigue resistance of SiN-GC scaffolds under dynamic loading at a much higher strain level than the cortical bone at the physiological condition, which is usually under 0.2% strain (Schaffler et al., 1990; Acevedo et al., 2018). The sinusoidal compression with a frequency of 1 Hz was used to mimic the load pattern during human locomotion (Morlock et al., 2001; Schreivogel et al., 2019). In line with results under static conditions, the scaffolds with higher SiN concentrations in the bioreactor also demonstrated higher mineralization rates and upregulation of osteogenic genes (Figures 7C–G). In addition, the osteogenic gene expression in all testing scaffolds under cyclic loading conditions was higher than that under static conditions (Supplementary Figure S5). It is due to the sensitivity of cells in their biomechanical environment that mechanical stimuli from cyclic loading work as a signal to bones which are naturally programmed to respond to repeated stimuli in the damage–repair process (Taylor et al., 2007; Acevedo et al., 2018). However, precise mechanisms on how cyclic loads and the specific loading condition induce the cellular responses remain unknown, and further studies are required to understand the specific role for each parameter in the load-bearing situation. Nevertheless, in line with previous studies, we were able to verify that the fatigue caused by cyclic loading induces cells in highly stressed scaffolds and osteoconductivity from SiN-GCs accelerated bone formation (Rath et al., 2008; Zhang et al., 2009). Additionally, there was no deformation found after the cyclic loading, which suggests extraordinary fatigue resistance of SiN-GC due to shape recovery property and sponge-like characteristics of cryogels even with SiN reinforcement (Kim et al., 2018; Lee et al., 2020). These allow SiN-GCs to be used as load-bearing scaffolds that require to withstand dynamic loading in the physiological system.

## Conclusion

In this study, we developed a SiN-GC cryogel system by loading SiN microparticles into a macroporous GC cryogel to fabricate a biomimetic scaffold with antibiofilm and osteogenic properties. Compared to GC, reinforcement with SiN was able to enhance the mechanical properties of the scaffold, which could provide a stable mechanical support in defect areas and an osteogenic environment for cells, while maintaining the benefits of GC such as a macroporous structure and well-interconnected porosity. In addition, negative charge and surface area increase due to the acicular polycrystal microstructure of SiN increased the apatite formation in SBF and protein adsorption capacity which would lead to faster bone formation. We were able to confirm that the SiN-GC group with higher SiN concentration resulted in stronger antibiofilm activity, higher cellular proliferation, higher mineralization, and osteogenic gene upregulation. Finally, we confirmed the enhanced osteogenic profile of the SiN-GC cryogel system even under cyclic compressive loading in a bioreactor. Based on these results, this study demonstrates a promising potential of SiN as a component in a biomaterial system and suggests the SiN-GC cryogel system as a new approach for bone tissue engineering.

## Data Availability

The raw data supporting the conclusion of this article will be made available by the authors, without undue reservation.
