# Supplementary material for: Silicon Nitride, a Bioceramic for Bone Tissue Engineering: A Reinforced Cryogel System With Antibiofilm and Osteogenic Effects
Source: Front Bioeng Biotechnol. 2021 Dec 15;9:794586. doi: 10.3389/fbioe.2021.794586 (PMC8714913; doi:10.3389/fbioe.2021.794586)
Supplement: Supplementary file 2 [file DataSheet1.docx]

Supporting Information for Publication

Silicon Nitride, A Bioceramic for Bone Tissue Engineering:

A Reinforced Cryogel System with Antibiofilm and Osteogenic Effects

Seunghun S. Lee^1,*^, Leanid Laganenka^2^, Xiaoyu Du^1^, Wolf-​Dietrich Hardt^2^ and Stephen J. Ferguson^1,*^

^1^ Institute for Biomechanics, Department of Health Sciences and Technology, ETH Zurich, Zurich, Switzerland

^2^ Institute of Microbiology, Department of Biology, ETH Zurich, Zurich, Switzerland

*Corresponding author at: ETH Zurich, Institute for Biomechanics

Hönggerbergring 64, HPP O24, 8093 Zürich, Switzerland

E-mail: seunglee@ethz.ch. Phone: +41 44 633 79 58.

E-mail: sferguson@ethz.ch. Phone: +41 44 633 93 305.

**Supporting Information Captions**

**Figure S1.** Planktonic culture from SiN-GC cryogels.

**Figure S2.** 2D *in vitro* test : cellular response to SiN microparticles.

**Figure S3.** 2D *in vitro* test : osteogenic effect of SiN microparticles.

**Figure S4.** Bioreactor setup for cyclic loading condition.

**Figure S5.** Relative fold induction of osteogenic genes under static condition or cyclic loading condition by the bioreactor.

**Supplementary Video 1.**

Cyclic monoaxial compressive loading of SiN-GC cryogels in a bioreactor. Cyclic loading was performed for 1 hour/day at a frequency of 1 Hz and strain of 10%.


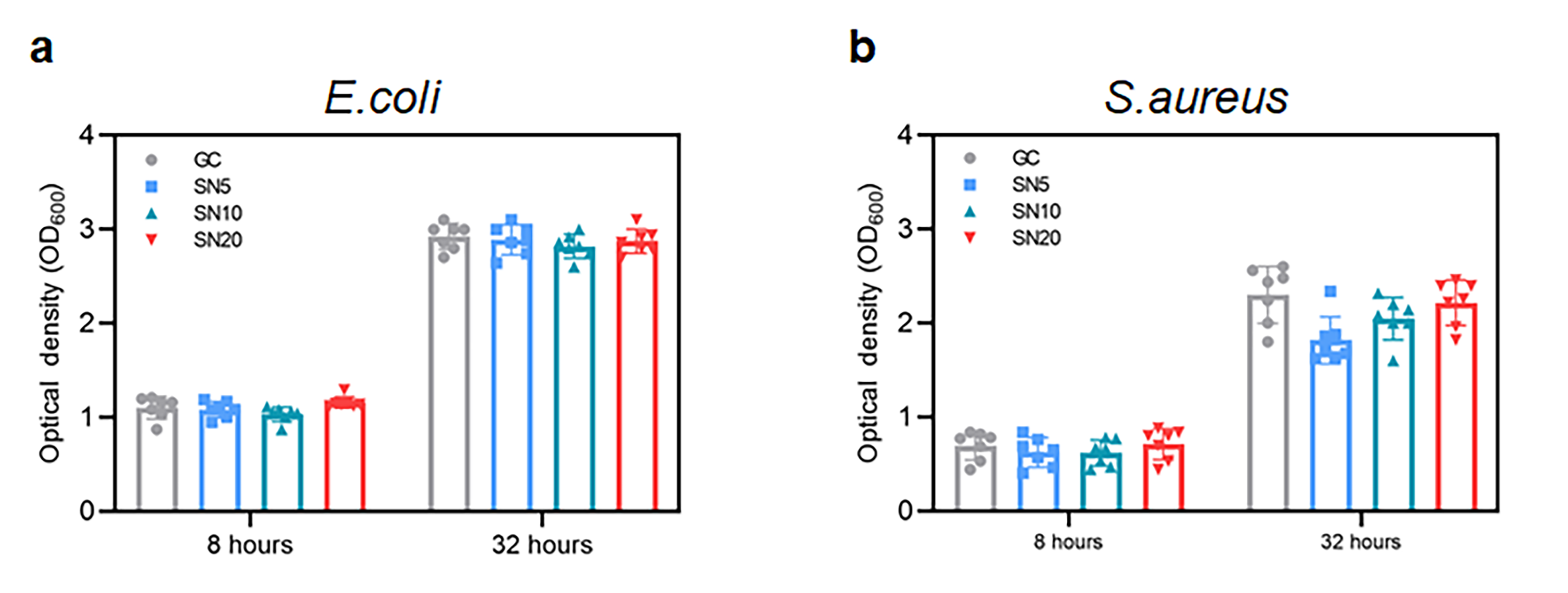


**Figure S1. Planktonic culture from SiN-GC cryogels. Planktonic culture of (a)** *E.coli* and **(b)** *S.aureus* from the media was measured after 8 and 32 hours of culturing on SiN-GC cryogels. Error bars indicate SD. n = 7.


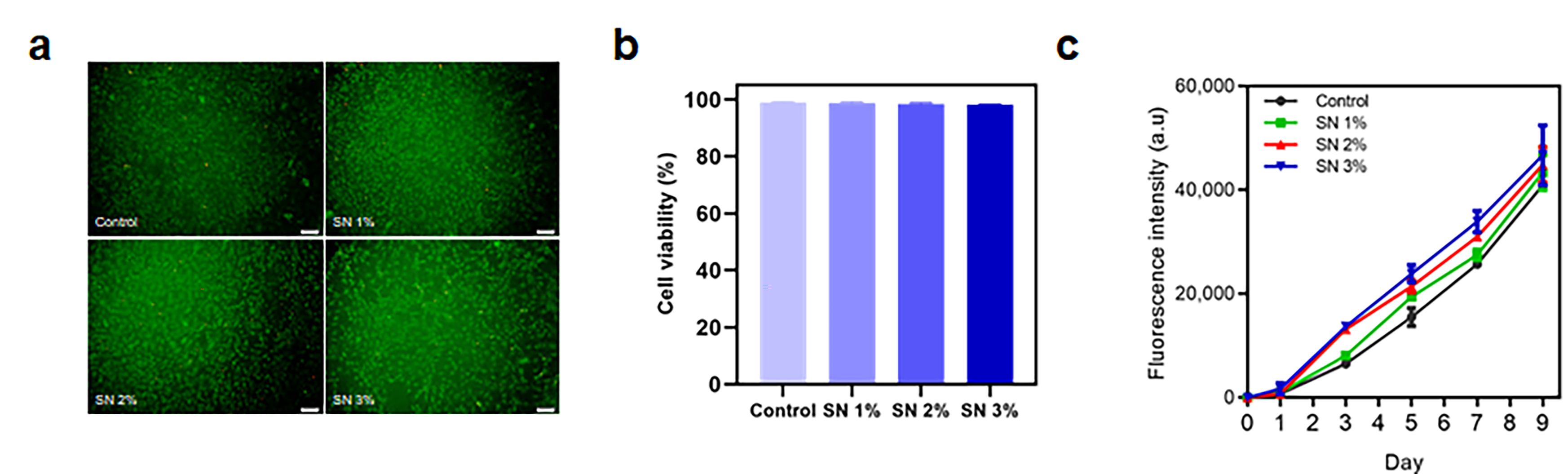


**Figure S2. 2D *in vitro* test : cellular response to SiN microparticles. (a)** Live/Dead staining of MC3T3-E1 pre-osteoblasts after 3 days culturing in conditioned media with different SiN concentrations. n = 4. scale bar = 100 μm. **(b)** Quantitative analysis of cell viability from live/dead assay **(c)** Proliferation rate of pre-osteoblasts culturing in conditioned media with different SN concentrations. 5×10^3^ of cells were seeded per well in 48 well plate and proliferation rate was analyzed by Presto blue assay. Error bars indicate SD. n = 3.


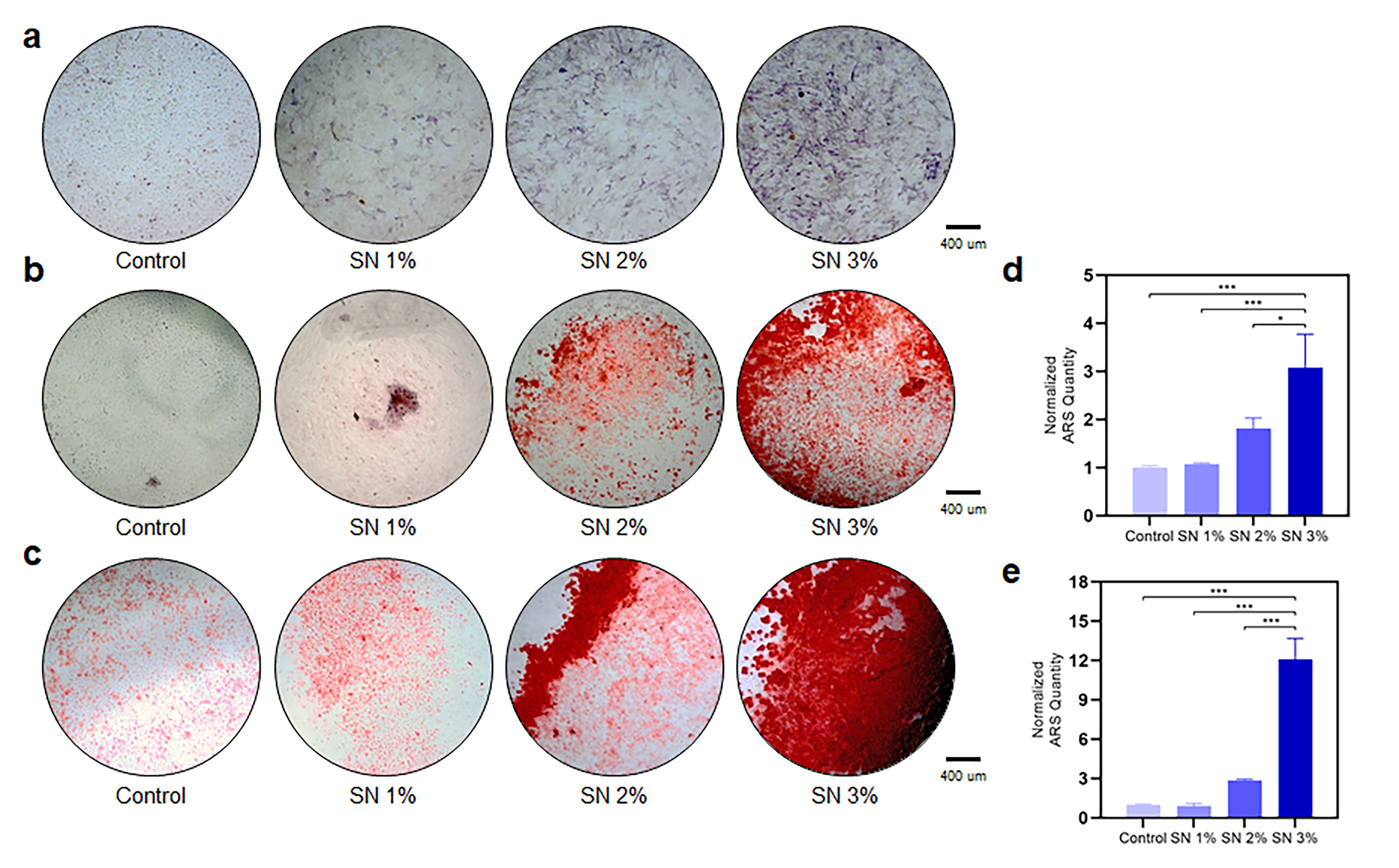


**Figure S3. 2D *in vitro* test : osteogenic effect of SiN microparticles. (a-c)** Representative images of **(a)** Alkaline phosphatase (ALP) staining after 7 days of osteogenic differentiation and **(b,c**) Alizarin Red S (ARS) staining after **(b)** 7 days and **(c)** 14 days of osteogenic differentiation of MC3T3-E1 in the osteogenic media with different SN concentration. Quantitative analysis of ARS staining on **(d)** day 7 and **(e)** day 14 was measured by utilizing cetylpyridinium chloride (CPC) method. Error bars indicate SD. n = 3.


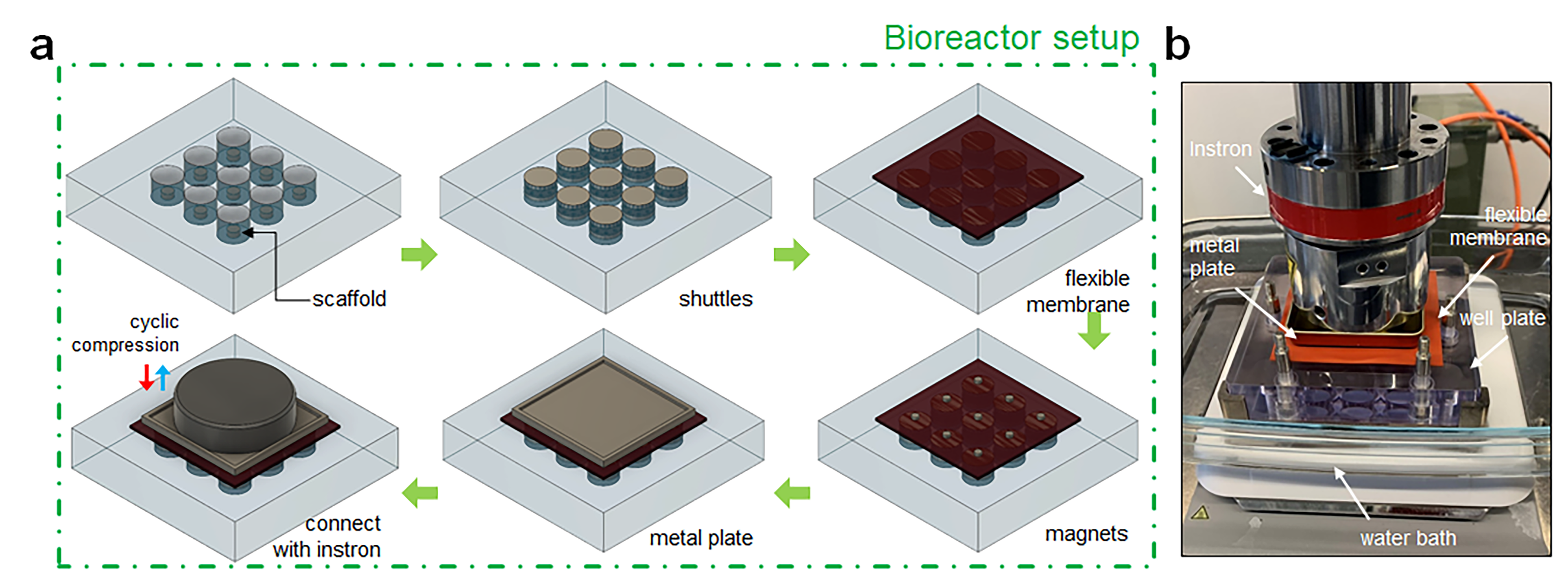


**Figure S4. Bioreactor setup for 3D culture under cyclic loading condition (a)** Schematic illustration of setup of the bioreactor that was used to apply cyclic loading during 3D culture. **(b)** Photograph of the bioreactor used in the study.


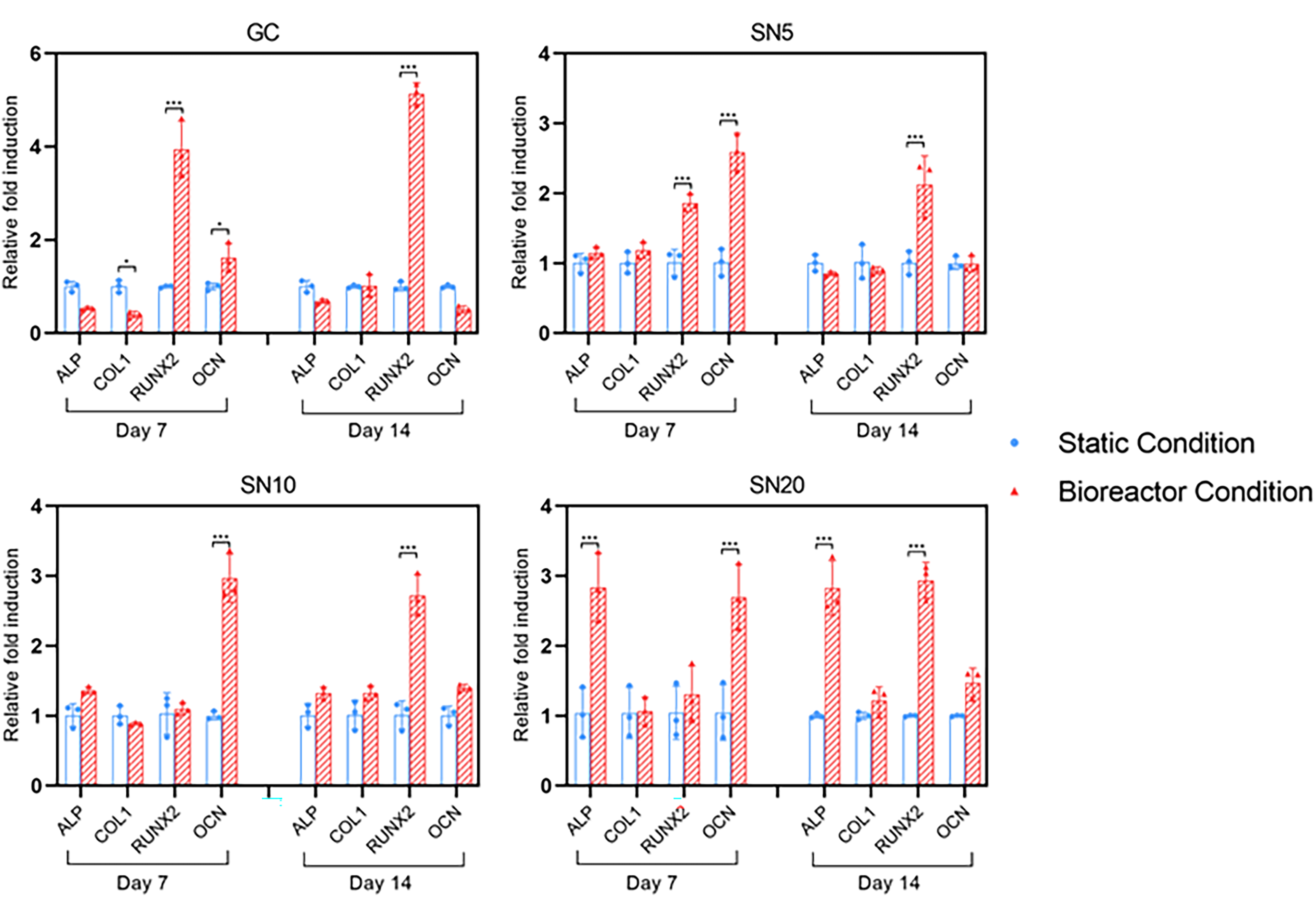


**Figure S5. Relative fold induction of osteogenic genes under static conditions or cyclic loading conditions in the bioreactor.** Pre-osteoblasts were seeded on the SiN-GC cryogels and cultured for 7 and 14 days with osteogenic medium under static conditions or cyclic loading conditions in the bioreactor (1 hour/day of cyclic compression at 1 Hz frequency and 10% strain). Error bars indicate SD. n = 3.
